# Landscape carbon trajectories after spruce budworm outbreaks in Canada’s Eastern boreal forest: effects of salvage intensity and wood-use pathways

**DOI:** 10.1007/s10980-026-02376-1

**Published:** 2026-06-06

**Authors:** A. Ameray, D. S. Pureswaran, J. Laganière, R. W. Buchkowski

**Affiliations:** 1 Canadian Forest Service, Atlantic Forestry Centre, Natural Resources Canada, 1350, Regent Street, Fredericton, NB E3C 2G6 Canada; 2 Canadian Forest Service, Laurentian Forestry Centre, Natural Resources Canada, 1055 du P.E.P.S, Stn. Sainte-Foy, Québec, QC G1V 4C7 Canada; 3 Department of Biology, University of Western Ontario, London, Canada; 4 Canalytics Group, Québec City, Québec Canada

**Keywords:** LANDIS-II, Forest management, Carbon dynamics, Salvage biomass, Harvested wood products, Net sector production, Transport emissions, Wood processing

## Abstract

**Context:**

Salvage biomass harvesting is increasingly recognized as a strategic approach to reduce emissions associated with spruce budworm (SBW) damage in eastern Canada by diverting biomass that would otherwise decompose in forests toward bioenergy and harvested wood products (HWP). However, its effects on overall system carbon dynamics remain poorly understood.

**Objective:**

We assessed how SBW-related salvage harvesting influences long-term (2010–2110) forest-sector carbon dynamics in the Côte-Nord region of Québec, Canada, considering net sector production (NSP), which is the sum of net carbon sequestration by ecosystem and HWP less operational emissions (harvesting, forwarding, transport, sawing, chipping, pelletization). This study evaluates the biophysical carbon balance and sector-level carbon dynamics of salvage harvesting, without accounting for substitution.

**Method:**

We used the LANDIS-II model, its Forest Carbon Succession extension, and additional modules to simulate wind, fire, and SBW disturbances under multiple climate pathways. A no-additional-salvage reference (S0) was compared to increasing salvage areas (S1–S3) of dead snag stems. All additional salvaged biomass was directed to one of three pathways: bioenergy, sawnwood with a 35-year half-life, or sawnwood with a 60 year half-life.

**Results:**

Salvage harvesting had minimal impact on ecosystem carbon pools but slightly reduced heterotrophic respiration by removing decomposing deadwood. The net carbon outcome depended strongly on product pathway: directing salvaged biomass to bioenergy resulted in immediate emissions and negative balance relative to the reference scenario; short-lived sawnwood (35-year half-life) increased HWP storage but accumulated decomposition emissions over time, also resulting in negative carbon balance; only long-lived sawnwood (60-year half-life) reduced total emissions sufficiently to approach/exceed carbon neutrality—and only under warmer climate scenarios (RCP4.5–RCP8.5) by late century. Operational emissions from harvesting, transport, and processing remained small relative to ecosystem and HWP carbon fluxes.

**Conclusions:**

Within the modeled system boundary (excluding substitution effects), the forest-sector carbon outcome of salvage harvesting depended strongly on target area, product type and their longevity; however, any benefits for reduced carbon emissions are likely to come from substitution effects unless warming dramatically increases decomposition and HWP have above-expected half-lives.

**Supplementary Information:**

The online version contains supplementary material available at https://doi.org/10.1007/s10980-026-02376-1.

## Introduction

The boreal forest is crucial to the global carbon cycle as it stores large amounts of carbon in both vegetation (88 PgC) and soils (460 PgC) (Pan et al. 2011), thereby contributing to climate change mitigation (Dixon et al. 1994; Gauthier et al. 2015). However, the balance within this ecosystem is increasingly threatened by natural disturbances, such as insect outbreaks and extreme meteorological events due to climate change, which can significantly alter forest capacity to sequester carbon and disrupt broader ecological processes (Dymond et al. 2010; Boulanger et al. 2017; Gauthier et al. 2023). In the last decade, the Canadian boreal forest has acted as a carbon source because of natural disturbances (ECCC 2023).

Insect outbreaks by native or invasive species present significant challenges due to their potential to spread synchronously over vast areas, leading to extensive tree mortality and profound impacts on the forest ecosystem carbon (Gauthier et al. 2015; Pureswaran et al. 2018). In North America, millions of hectares are defoliated annually by insect outbreaks, with the spruce budworm (SBW; *Choristoneura fumiferana*) being the most prominent in eastern Canadian boreal forests (MacLean 2016). The severity and spread of SBW outbreaks are influenced by factors such as the age and abundance of host species, as well as specific bioclimatic conditions like temperature and precipitation, meaning that SBW outbreaks will interact with climate change, fire regime, and management to determine the future of the eastern boreal (Boulanger et al. 2017; Pureswaran et al. 2019).

The consequences of SBW outbreaks extend beyond the immediate loss of forest cover; they also result in reduced economic returns due to lower harvest volumes and disruptions to the carbon cycle through decreased net primary productivity (NPP) and increased heterotrophic respiration (*R*_h_) (Dale et al. 2001; Dymond et al. 2016). These disturbances lead to higher carbon emissions depending on their severity, particularly from fires that are exacerbated by the increased availability of fuel produced by insect outbreaks (Dymond et al. 2010; Boulanger et al. 2014). The recent SBW outbreak in Quebec, which began in 2006 and by 2022 had defoliated over 12 million hectares, illustrates the persistent and severe nature of these infestations and their potential to transform forest landscapes from carbon sinks into carbon sources (Dymond et al. 2010; MRNF 2023).

Salvage biomass harvesting has emerged as a potential strategy for reducing carbon emissions following SBW outbreaks in eastern boreal forests. Recognized by the Intergovernmental Panel on Climate Change (IPCC), salvage harvesting can support local industries and increase the biomass used in harvested wood products (HWP) such as bioenergy, sawnwood, and pulp and paper (Smyth et al. 2014; Laganière et al. 2017; Lee et al. 2023). These wood product categories store carbon for varying durations—long-term in sawnwood and short to medium-term in pulp and paper products (Pingoud et al. 2003), contributing to enhancing the carbon sequestration and storage potential of Canada's forest sector if wood resources are used more efficiently or substituted for other emissions (Smyth et al. 2014). Salvage harvesting is part of better utilization of forest products, but it remains unclear whether incentivizing salvage harvesting after SBW defoliation is beneficial if it leads to additional biomass removal rather than offsetting the harvesting of live trees (Smyth et al. 2014; Laganière et al. 2017). Despite growing interest in salvage harvesting, a critical knowledge gap remains: the net carbon balance of salvage operations—integrating ecosystem-level carbon dynamics, supply-chain emissions, and HWP carbon cycle—has not been comprehensively quantified under varying climate futures. Previous studies have examined individual components of this system, but few have coupled spatially explicit forest landscape models with full life-cycle assessments of HWP pathways to evaluate whether salvage harvesting produces a positive or negative carbon balance across the forest sector.

This research aims to explore how salvage biomass after SBW affects carbon dynamics in the eastern boreal forest by examining ecosystem carbon balance, operational emissions (harvesting, forwarding, sawing, chipping, and pelletization), and sequestration across different wood product pools used in the eastern boreal forest (sawnwood, pulp and paper, and bioenergy). We do not account for substitution effects in the bioenergy and HWP supply chain, hence underestimating the benefits of salvage harvesting. Under various climate change scenarios (Baseline, RCP2.6, RCP4.5, and RCP8.5), this study investigates: 1) the impact of different salvage biomass scenarios post-SBW outbreaks on carbon dynamics (fluxes and stocks) using the LANDIS-II model, 2) the life cycle assessment (LCA) of all HWP categories and their capacity to store carbon in the short and long term, 3) forest biomass processes including machinery emissions during harvesting, forwarding, transport to storage facility and transformation related carbon emissions (sawing, chipping, and pelletization), and 4) the forest sector's carbon balance dynamics by calculating the net sector production (NSP), which considered the net biome productivity (NBP) in the ecosystem, the processing operations, and the carbon remaining in HWP pools after accounting for combustion and decomposition. This article seeks to provide an estimate of the role of salvage biomass as a sustainable strategy within the context of wood production in the eastern boreal forest and its potential contribution to altered carbon storage patterns and sector-level carbon dynamics (Laganière et al. 2017; Pureswaran et al. 2018; Boulanger et al. 2023). It is important to note that without accounting for substitution effects (i.e., displacement of fossil fuels or carbon-intensive materials), our analysis quantifies carbon redistribution and the timing of emissions rather than full climate mitigation potential.

## Method and materials

### Study area

**Fig. 1:**
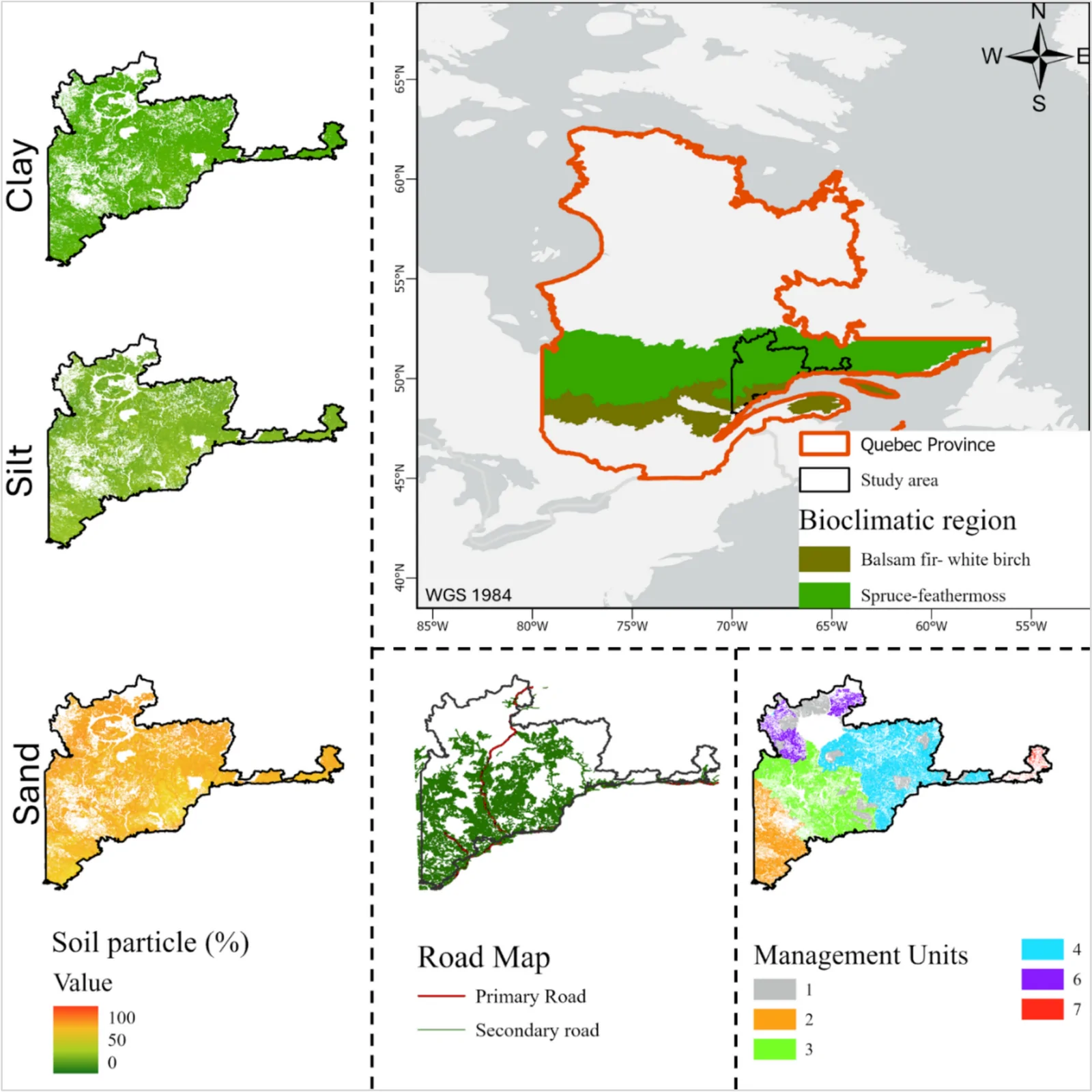
Study area, road map, management units, and soil particle percentage in the study area used to classify soil texture

The study was carried out in the Côte-Nord, an administrative region in the province of Quebec in Canada. It is located in the spruce–feathermoss and balsam fir–white birch bioclimatic domains of the Boreal Shield near the northern commercial forest limit (Fig. 1). Beyond this limit, forests are not managed for timber production because of low productivity (Jobidon et al. 2015). The region experiences an average annual precipitation of approximately 1000 mm, with mean annual temperatures ranging from -2.0°C in the northern ecoregions to 2.2°C in the southern ones (Wang et al. 2016). The landscape is dominated by black spruce (*Picea mariana*), balsam fir (*Abies balsamea*), jack pine (*Pinus banksiana*), white birch (*Betula papyrifera*), trembling aspen (*P. tremuloides),* and other species (MRNF 2010). The current dominant age class is 100—200 years old. The dominant soil texture is sandy-loam (Duchesne and Ouimet 2021; Ameray et al. 2023a). We identified and excluded water bodies, wetlands, islands, and non-commercial species from our simulation to focus our analysis on areas where salvage harvesting could be conducted.

## Simulation models

The modeling framework couples the LANDIS-II forest landscape model with carbon accounting modules to simulate forest dynamics under various climate and management scenarios. The workflow incorporates the following components: (1) forest growth and succession, (2) natural disturbances such as spruce budworm, fire, and wind, (3) harvest and salvage operations, (4) carbon tracking in harvested wood products, and (5) operational emissions from the supply chain. This integrated approach facilitates the calculation of net sector production (NSP) across all scenarios. Each component is described in the following subsections.

## Model description

### Forest succession and growth

**Fig. 2:**
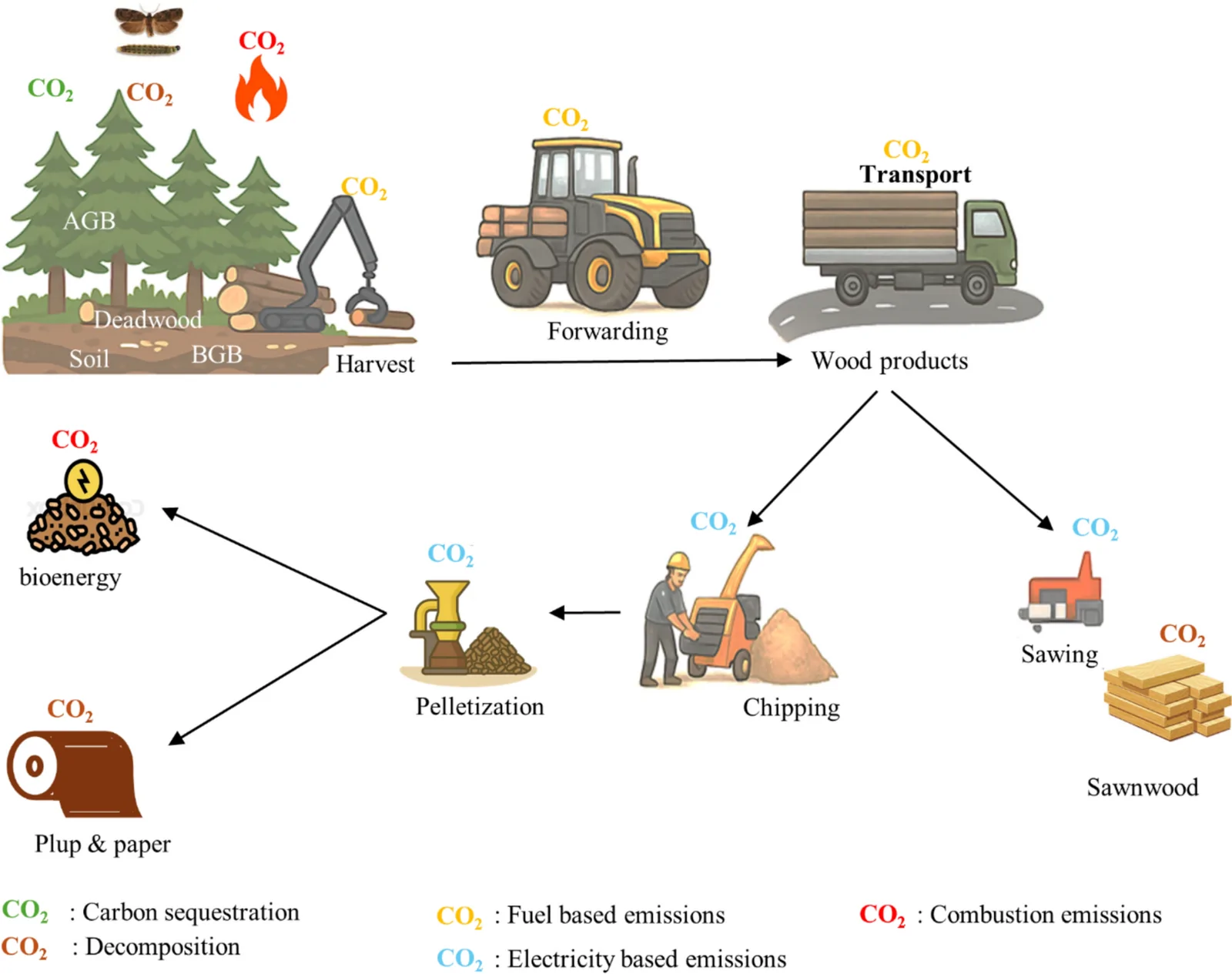
Framework linking LANDIS-II ecosystem carbon dynamics with supply-chain and harvested-wood-product (HWP) carbon accounting. NPP adds carbon to biomass and soil; losses occur via Rh, disturbances (SBW, fire), and harvest. Harvested biomass generates emissions from forwarding, transport, and processing (sawing, chipping, pelletization) and is allocated to HWPs (sawnwood, pulp and paper, bioenergy), which release carbon through decomposition or combustion over time

We used LANDIS-II (V7.1), a stochastic, spatially explicit forest landscape model, to run landscape-scale simulations across the Côte-Nord region at a spatial resolution of 200 × 200 m (4 ha) (Fig. 2) (Scheller et al. 2007). This model simulates succession, cohort growth, dispersal, as well as various disturbances such as management, wildfires, windthrow, and insect outbreaks, along with other degenerative processes like senescence and mortality (Mladenoff and He 1999; Scheller and Mladenoff 2004). LANDIS-II simulates forest dynamics across large spatial and temporal scales by accounting for neighborhood interactions among landscape cells, including adjacent processes (e.g., fire spread) and non-adjacent processes (e.g., seed dispersal) (Mladenoff and He 1999). Seed dispersal is modeled using Ward’s negative exponential algorithm with species-specific dispersal distances (Ward et al. 2005).

The LANDIS-II model incorporates multiple sub-models (extensions) to assess forest succession and growth, and disturbances. The selection of a particular sub-model over another is dependent upon the available data and the project's specific objectives. We used the extensions Forest Carbon Succession (ForCS) V3.1 (Dymond et al. 2016), Base Fire V4.0 (Scheller and Domingo 2018), Biomass Harvest V4.4 (Gustafson et al. 2000), Base Biological Disturbance Agent V4.1 (Sturtevant et al. 2004), and Base Wind V3.1 (Scheller et al. 2018).

The ForCS extension in LANDIS-II is designed to calculate the reproduction, aging, growth, and mortality of tree cohorts, as detailed in Dymond et al. (2016). Biomass carbon accumulation resulting from growth and reproduction aligns with the Biomass Succession (v5.7) extension and methods specified in Scheller and Mladenoff (2004). Additionally, ForCS simulates the trend of forest stands and carbon dynamics, including carbon turnover, net growth, NPP, *R*_h_, net ecosystem productivity (NEP), NBP, transfers between pools, losses from logging (carbon transfers to HWP), and carbon emissions from decay or combustion (Dymond et al. 2016; Hof et al. 2017).

As outlined by Dymond et al. (2016), ForCS incorporates dead organic matter (DOM) and soil dynamics based on the Carbon Budget Model of the Canadian Forest Sector (CBM-CFS3) model (IPCC Tier 3 approach). Default parameters from CBM-CFS3, particularly for DOM carbon transfers and disturbance matrices were used (Kurz et al. 2009). We applied the updated decay rates for softwood and hardwood groups (Table A1), as provided by Hararuk et al. (2017). These rates were specifically calibrated for the CBM-CFS3 model using data from the National Forest Inventory across Canada. The model also accounted for the direct impact of temperature on DOM, with temperature inputs adjusted for each climate scenario and time period. We used a spin-up approach to initialize the soil pools (see ForCS user guide for more details). Before starting the simulation, we confirmed that the initial simulated total soil carbon pools matched literature values (60—100 tC ha^−1^) (Paré et al. 2011a).

ForCS requires species establishment probability (*Pest*), maximum aboveground net primary production (*MaxANPP*), and maximum aboveground biomass (*MaxAGB*) as inputs (Table A2). We used the PnET-II succession model (Photosynthesis and EvapoTranspiration) to simulate *Pest*, *MaxANPP*, and *MaxAGB* separately. We had previously calibrated and validated this model using ForCS in the same study area, incorporating yield curves from forest inventories and remote sensing data from MODIS and Sentinel-2 images (Ameray et al. 2023a, 2024). For the baseline climate scenario, we derived these the three parameters from annual weather data, using the historical monthly time series from 1991 to 2010 and assuming a constant atmospheric CO_2_ concentration of 389 ppm (the 2010 value) based on the Representative Concentration Pathway (RCP) database. For the RCP climate change pathways, we recalculated and updated the three parameters for each climate scenario (RCP2.5, RCP4.5, and RCP8.5) across three time periods: 2010–2040, 2041–2070, and 2071–2110.

### Landscape initial conditions and climate

The species and age data, representing the initial communities mapped at a 200 m resolution, were obtained from the 2010 spatial forest inventory maintained by Quebec’s Ministère des Ressources Naturelles et des Forêts (MRNF 2010). In the LANDIS-II model, each species was linked to life-history traits that govern its regeneration and dispersal, including longevity, age of sexual maturity, shade tolerance, fire tolerance, seed dispersal distance, sprouting ability, and post-fire regeneration characteristics, as compiled from previous studies (Table A3) (Boulanger et al. 2017; Ameray et al. 2023a). Additionally, the LANDIS-II model requires the landscape to be divided into specific ecoregions, or land types, where soil and climate conditions are assumed to be uniform within cells, with distinct growth and reproduction functions for each land type. The ecoregions used in this study were defined using the soil map developed by Duchesne and Ouimet (2021) and the bioclimatic regions from the MRNF geodatabase (Fig. 1).

For climate data, we used historical monthly weather records for each RCP scenario (RCP2.6, RCP4.5, RCP8.5) from the ClimateNA model, which is based on CanESM2 projections (Figure A1). This local downscaling model provides climate data tailored to specific locations in North America (latitude, longitude, and elevation) (Wang et al. 2016). The model incorporates multiple General Circulation Models (GCMs) to cover paleoclimatic conditions, historical data from 1901 to 2010, and future projections (Wang et al. 2016). To represent the climate of each ecoregion, we averaged climate parameters across 10 randomly selected points within each. For the current baseline climate, we used historical monthly climate data from 1991 to 2010, ensuring a stable baseline climate. The CanESM2 projections indicate that by 2100, mean annual temperatures will rise by approximately 3°C, 5°C, and 7.5°C for RCP2.6, RCP4.5, and RCP8.5, respectively, relative to the baseline (Ameray et al. 2024). Additionally, precipitation is expected to increase by about 40 mm under RCP2.6 and by over 100 mm under both RCP4.5 and RCP8.5 scenarios.

## Natural disturbances

### Spruce budworm

The insect outbreak modeling in this study used the Biological Disturbance Agent (BDA) extension (Sturtevant et al. 2004). It operates by assessing the probability of an outbreak based on forest stand characteristics (Table A4), including age, species composition, and spatial distribution, which influence vulnerability to insect attacks. By simulating SBW life cycle and its interactions with host species, the model predicts tree mortality, which subsequently impacts forest carbon dynamics through changes in carbon stocks and fluxes. We relied on a 400-year dendrochronological reconstruction of SBW outbreaks in southern Quebec to set an average of 32 years between SBW outbreaks (Boulanger et al. 2017; Navarro et al. 2018). The defoliation started in 2006, so we assumed that mortality begins at time step 1 (2011) in our simulation because mortality lags behind defoliation.

The BDA model extension simulates mortality, which we calibrated using annual and cumulative areas affected by mortality due to SBW, based on historical aerial photographs provided by the Quebec Ministry of Forests (Figure A2) (MRNF 2010; 2023). This dataset includes crucial data on the annual area impacted by SBW within the simulated landscape from 1967 to 1988. We processed raster data for each year to monitor changes in forest mortality and match annual and cumulative proportions of affected area and tree mortality, which was key to fine-tuning the BDA model (Figure A2,A3).

### Fire and winds

The fire and wind disturbance models in this study expanded previously calibrated and parameterized extensions, as detailed in our earlier works (Ameray et al. 2023b) in the same study area. The Base Fire extension simulates fire regimes through stochastic fire events, driven by factors such as fire ignition, initiation, and spread within each ecoregion. The model uses input data, including ignition probability, fire region maps, and fire size (minimum, mean, and maximum), as well as fire severity to simulate these events (Scheller and Domingo 2018). Given the strong influence of climate on wildfire regimes, we calibrated the burn rate, representing the percentage of land disturbed annually, for each ecoregion under each climate change scenario, including the current baseline (Table A5), drawing from established literature (Bergeron et al. 2006; Boulanger et al. 2014). Similarly, the Base Wind extension was employed to stochastically simulate windthrow disturbances, and generally has a smaller impact on boreal forests than fire or insect outbreaks. This extension models windthrow based on parameters such as intensity, size, spread, severity, and rotation period (Scheller et al. 2018). For calibration, windthrow size and period per ecoregion were parameterized using historical data from the forest inventory geodatabase (1970–2010) (Table A6). On average, windthrow affects about 0.0255% of the area annually in Quebec (Bouchard et al. 2009). The model calibration and parameterization relied on forest aerial surveys and published works in similar landscapes.

### Harvest and salvage biomass

The biomass harvest extension was used to simulate existing forest harvesting. This extension stochastically models the effects of different harvest treatments or prescriptions (e.g., clear cuts, partial cuts, selective cutting, salvage harvesting) and planting regimes. Following previous models for the same region (Ameray et al. 2024), 95% of the harvested area was managed using clear-cutting systems, with only 5% using partial cuts. The model requires the landscape to be divided into management areas, where stands are selected for harvesting based on species and age criteria, and the sequence in which they will be harvested is defined. The current management scenario (reference) is calibrated to reflect existing harvest rates for each prescription, which are typically 70% of the allowable annual cut (AAC) in the region. Only stems (merchantable wood) were collected in the study area and transferred to forest sector products. The harvest treatments and AAC used were reported by the Chief Forester of Quebec for each management area.

The existing biomass harvest extension in LANDIS-II did not support salvage logging following biological disturbances such as spruce budworm (SBW) outbreaks. To overcome this limitation, an external salvage biomass model was developed based on the CBM-CFS3 framework, employing the same equations and parameters as those used in the ForCS model. This approach differentiated between two pathways for dead snag stem biomass resulting from SBW–induced mortality: salvaged and non-salvaged. Salvage harvesting was applied only to grid cells with a minimum threshold of 5 tC ha⁻1. When snag stems were not salvaged, we tracked their fate as if they remained in the ecosystem. Their carbon was gradually released through processes governed by CBM-CFS3 assumptions (Kurz et al. 2009), including temperature-dependent decomposition, fire-induced emissions, and physical transfers to more stable pools (i.e., medium and slow). The decomposition rate was modulated using a Q₁₀ relationship based on mean annual temperature, while wildfire events emitted a fraction of the remaining carbon from each pool (Fast, medium, slow), depending on fire severity. Conversely, when snag stems were salvaged, the carbon was transferred to HWP pools, specifically for coniferous species (i.e., SBW host species), following the same assumptions applied to HWP carbon dynamics described in Sect. 3.2. The salvage harvesting targeted only the dead snag stem biomass (referred to as the "only-stem" harvest method, the predominant operational practice in the eastern Canadian boreal forest) (Sage et al. 2019), while all other woody debris was transferred to the DOM pools. The salvage areas tested, ranging from 1,000 to 4,000 ha yr⁻1, were chosen to reflect operational realities and current policy targets in the region, as defined in the MNRF special management plans (Table 1) (MRNF 2025).

**Table 1:** Tested scenarios description

Scenario (j)	Description	Salvage biomass harvested area(ha)
S0	Reference management scenario, where all the disturbances are considered, as well as the current harvest treatments without salvage biomass	0
S1	Other scenarios, where we added area for salvage biomass of dead snag stems following SBW outbreaks to S0	1000
S2		2000
S3		4000

Our salvage biomass scenarios S1 to S3 add additional harvest area from the affected stand by SBW to the reference

### HWP life cycle assessment

To quantify carbon dynamics associated with harvested wood products (HWP), we followed the IPCC framework, which tracks product carbon stocks over time using first-order decay (*k*) functions parameterized by product-specific half-lives (Eq. 1). For the reference scenario (S0), harvested biomass was allocated to product categories using Quebec’s average product mix: for softwoods, 42% sawnwood, 46% pulp and paper, and 12% bioenergy; for tolerant and intolerant hardwoods, 88% pulp and paper and 12% bioenergy (Beauregard et al. 2019). Product half-lives under S0 were taken from IPCC guidelines: 35 years for sawnwood, 2 years for pulp and paper, and 1 year for bioenergy. Bioenergy was assumed to result in immediate carbon release upon use (*k* = 1), consistent with its short-lived lifecycle. For salvage scenarios (S1–S3), only the additional biomass harvested following spruce budworm outbreaks relative to S0 was reallocated. Three wood-use pathways were tested: (i) 100% bioenergy, (ii) 100% sawnwood with a 35-year half-life, and (iii) 100% sawnwood with a 60-year half-life. The 60-year half-life was selected based on published HWP carbon stock studies applying first-order decay to long-lived products, and to approximate the expected residence time of SBW-killed DOM under baseline conditions. This choice allowed a direct comparison between retaining carbon on site versus transferring it to long-lived HWP pools under different climate and disturbance regimes.

For modeling purposes, we assumed that the wood product reservoirs were empty at the start of the simulations (time 0), and only the products generated during the simulations were considered. This approach enabled us to calculate the accumulation of remaining carbon (Cr_(t,i,j)_) at time (t) for each climate pathway (i) and salvage biomass scenario (j) by subtracting the emissions from bioenergy and the emissions due to the decomposition of sawnwood and pulp and paper products (Eq. 1). Cr_(t,i,j)_ reflects the net carbon accumulation in HWP over time after accounting for emission and decomposition. Our approach aligns with the IPCC Tier 2 suggestion, where decay is applied to the previous year's remaining stock (t—1) and the current (t) year’s inflow. The net flux of carbon remaining in HWP (ΔCr) was calculated as the variation between two consecutive years.

1 $$Cr_{{\left( {t,i,j} \right)}} \; = \;Cr_{{\left( {t - 1, i, j} \right)}} + V_{{\left( {t,i,j} \right) }} - E_{{\left( {t,i,j} \right)}}$$

where.

*Cr*_*(*t,*i,j*):_ Total carbon stock in year (*t*) for a given climate (*i*) and management scenario *j*, *Cr*_(t-1,*i,j*)_: Remaining carbon stock from the previous year (*t*—1). *V*_(t,*i,j*)_: Value added to the stock in year t (input of harvested fluxes). *E*_(t,i,j)_: Emissions from decomposition or burning at year *t*, calculated for each wood category.

## Transport and processing operation emissions

### Transport

We developed a transport model to calculate the transport distances for harvested materials. We employed a k—d tree structure for efficient nearest-neighbor searches and integrated a pathfinding algorithm to identify the shortest paths between critical points in the network, using *networkx* library in *python* (Platt 2019). This approach enabled us to calculate the distance (D1) from the nearest road cell (point 1) to a harvested cell, the distance (D2 ~ constant) from the nearest road cell (point 2) to a storage facility, and the shortest path (D3) between point 1 and point 2. We then determined the total transportation distance (Dtotal) from each harvested cell to the storage facility by summing D1, D2, and D3—the distance along the shortest path. For each harvested cell, the transport emission (*T*_(t,i,j)cell_) after collecting harvested biomass for each cell was calculated using Eq. 2 under different climate pathways (i) and management scenarios (j). Dtotal was doubled to account for a round trip and multiplied by the required number of trucks (n) to transport the harvested biomass from each cell, along with an emission factor (*f*) based on default values from the IPCC guidelines (Eq. 2). The number of trucks (n) was calculated based on a carrying capacity of 40t (Serra et al. 2016), and the harvested biomass average per prescription (silvicultural treatment) applied at each cell. For each management scenario (j) and climate change pathway (i), we calculated the total emissions (T_(t,i,j)_) per year (t) as follows (Eq. 2):

2 $$T_{{\left( {t,i,j} \right)}} \; = \mathop \sum \limits_{cell = 1}^{cell = n} \left( {Dtotal_{{\left( {t,i,j} \right)cell}} \times 2} \right) \times n_{{\left( {t,i,j} \right)cell}} \times f$$

### Other operations

We quantified process-related greenhouse-gas emissions for the five main stages of a mechanised boreal-forest supply chain—harvesting (*H*), forwarding (*F*), sawing (*S*), chipping (*C*) and pelletization (*P*) (Table A.7, Fig. 2)—within a life-cycle assessment framework. Fuel-intensive field operations (harvesting and forwarding) were parameterised with volume-specific diesel-use data reported for modern cut-to-length systems (Kärhä et al. 2024). The other operations (sawing, chipping, and pelletization) were characterised with energy-based emission factors synthesised from Canadian LCA studies (Lamers et al. 2014; Laganière et al. 2017), and detailed industry energy audits (Meil et al. 2009). All emissions coefficients of each stage were normalised by wood densities for coniferous and broadleaved species. These stage-specific carbon-intensity coefficients were applied to scenario-specific biomass flows (coefficients × processed biomass per operation) and summed with transport emissions to produce total processing footprints for each management (j), climate pathway (i), and time step (t) (Eq. 3).

3 $$Operation_{{\left( {t,i,j} \right)}} \; = \;T_{{\left( {t,i,j} \right)}} + H_{{\left( {t,i,j} \right)}} + F_{{\left( {t,i,j} \right)}} + S_{{\left( {t,i,j} \right)}} + C_{{\left( {t,i,j} \right)}} + P_{{\left( {t,i,j} \right)}}$$

## Data analysis

To account for variability among simulations, we repeated each salvage biomass scenario three times for each climate change pathway, resulting in a total of 48 simulations. Firstly, the total of carbon fluxes (NPP, *R*_h_, NEP, NBP; MtCO_2_eq yr^−1^) and pools (biomass and soil; MtCO_2_eq yr^−1^) were assessed at the ecosystem scale. Secondly, we quantified the absolute NSP (MtCO_2_eq yr^−1^) at year (*t*) by combining the estimated NBP_(t,i,j)_ from LANDIS-II outputs of each climate scenario (*i*) and salvage biomass strategies (*j*), the net carbon flux from harvested wood products (ΔCr_(t,*i,j*)_), and the carbon emissions associated with operations (*Operation*_(*t,i,j*)_) (Eq. 4). Thirdly, to assess the impact of salvage biomass scenarios (Table 1), we also calculated the magnitude of change in each variable (fluxes/pools) as the difference (Δ_(t,*i,j*)_) between each salvage biomass scenario (*V*_t,i,j_) and the reference scenario (*V*_t*,i*,1_) for each climate projection (*i*) (Eq. 5). This delta (Δ_(t,*i,j*)_) present the magnitude of change between different salvaged biomass scenarios and the reference scenario. Additionally, to identify the impact of salvaged biomass use (bioenergy vs. sawnwood) on the total carbon budget, the cumulative NSP (NSPa_(t,*i,j*)_) was quantified. The NSPa_(t,i,j)_ evaluates how bioenergy vs. sawnwood use influences the total carbon budget; the NSPa difference between S0 and S1–S3 gives the direct effect of salvage strategies on the forest sector’s carbon budget. Although LANDIS-II is a spatially explicit, we report landscape-aggregated dynamics rather than spatial patterns, consistent with ForCS-based accounting studies (Dymond et al. 2016; Boulanger et al. 2017; Hof et al. 2017).

4 $$NSP_{{\left( {t,i,j} \right)}} \; = \;NBP_{{\left( {t,i,j} \right)}} + \Delta Cr_{{\left( {t,i,j} \right)}} - Operation_{{\left( {t,i,j} \right)}}$$

5 $$\Delta_{{\left( {t,i,j} \right)}} \; = \;V_{{\left( {t,i,j} \right)}} - V_{{\left( {t,i,1} \right)}}$$

Model outputs at year 0 were validated against empirical aboveground biomass from the 4th National Forest Inventory (*n* = 2237 plots) (MRNF 2010). Agreement was assessed using landscape-scale and land-type-stratified mean comparisons, with uncertainty quantified via bootstrap 95% confidence intervals. Simulated mean biomass closely matched observations (Δ =  − 1.6 t ha⁻1), indicating no systematic bias at the regional scale. However, an RMSE of 35.7 t ha⁻1 and quantile discrepancies indicate that the model reproduces mean trends but underestimates local variability. Bias was spatially heterogeneous, with the largest departures concentrated in northern land types (Figure A4).

## Results

### Salvage biomass effect on carbon dynamics at the ecosystem scale

#### Carbon dynamics under reference management scenario

##### Carbon pools

**Fig. 3:**
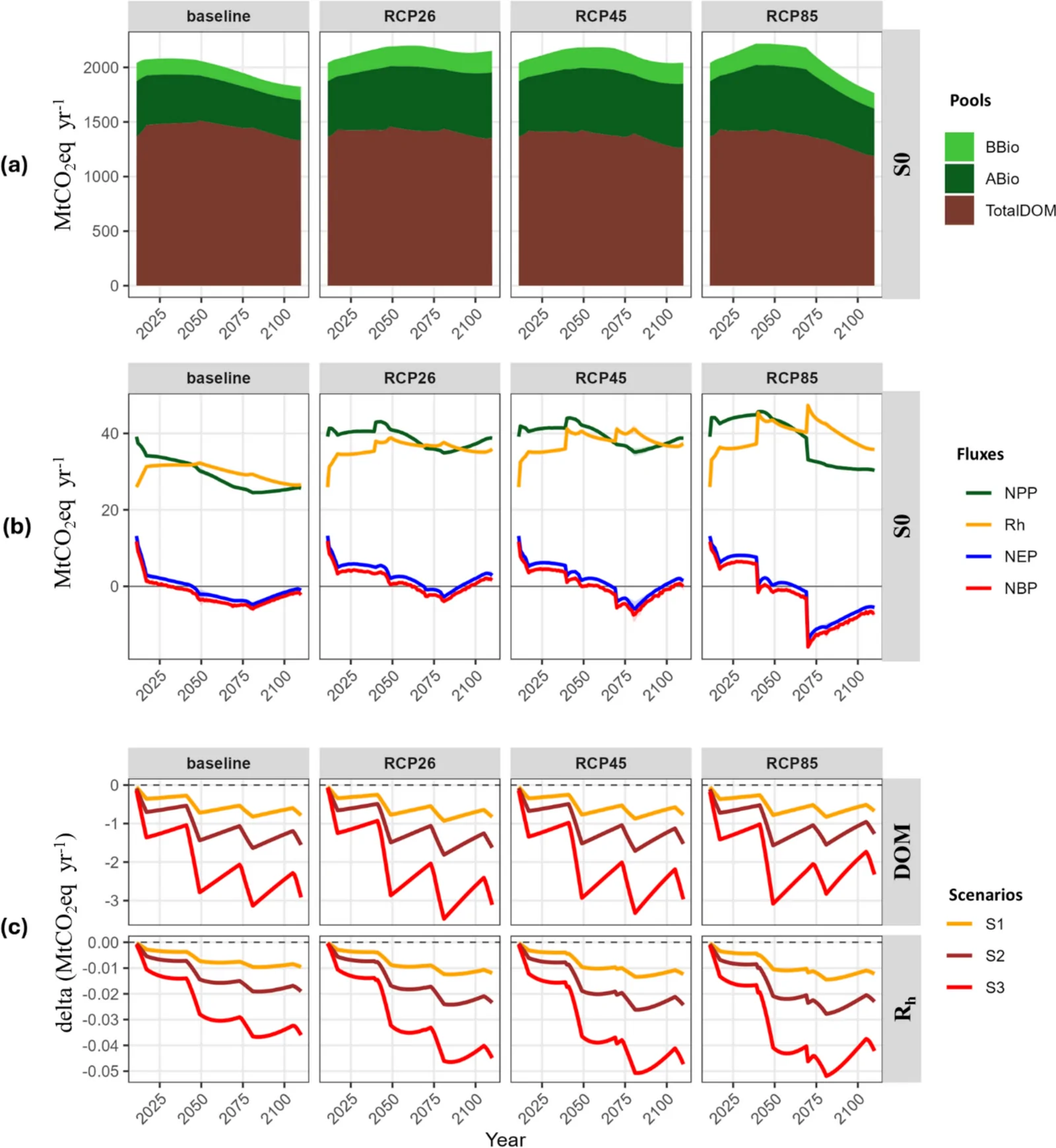
Climate and salvage effects on ecosystem carbon under four climate pathways (baseline, RCP2.6, RCP4.5, RCP8.5). **a** Carbon pools—dead organic matter (DOM), aboveground biomass (ABio), and belowground biomass (BBio)—decline after 2050 under baseline climate, with pronounced losses under RCP8.5 post-2070. **b** The forest transitions from carbon sink to source as heterotrophic respiration (*R*_h_) exceeds net primary productivity (NPP); c (NEP = NPP − *R*_h_) and net biome productivity (NBP) turn negative after successive SBW outbreaks. **c** Salvage harvesting (S1–S3) reduces DOM stocks and *R*_h_ emissions relative to reference (S0), with greater reductions under warmer scenarios. See Figure A.7 for NEP and NBP deltas

Under the baseline climate and S0, total carbon storage across the landscape—comprising biomass and soil—remained relatively stable at approximately 1800 MtCO_2_eq yr⁻1 until 2050, after which it declined to about 1700 MtCO_2_eq yr⁻1 by 2100 (Fig. 3 (a)). This decline closely aligned with a decrease in the area of older black spruce stands (> 120 years), which dropped by nearly 15% after 2050, as shown by the age structure analysis (Figure A5). This decrease was particularly evident in the DOM pool—which includes carbon in deadwood, litter, humus, and mineral soil—and was likely driven by elevated *R*_h_ rates (Fig. 3 (b)). Across all climate change scenarios, carbon stocks in biomass and soil initially increased between 2010 and 2070 by 10–15%, possibly due to enhanced productivity of certain species, mainly black spruce under RCP2.6 and RCP8.5 (Figure A6). However, under RCP8.5, a pronounced reduction in total carbon stocks of approximately 18% was observed after 2070 due to increased disturbance frequency and elevated decomposition rates (Fig. 3 (b)).

##### Carbon fluxes

Under the baseline climate and S0 scenario, the total landscape NPP began at approximately 39 MtCO_2_eq yr⁻^1^ in 2010 and declined to around 33 MtCO_2_eq yr⁻^1^ during the first spruce budworm (SBW) outbreak period (2010–2017) (Fig. 3 (b)). This decreasing trend continued, reaching 29 MtCO_2_eq yr⁻^1^ by the end of the simulation in 2110. In contrast, *R*_h_—representing carbon dioxide emissions from decomposition—started at about 26 MtCO_2_eq yr⁻^1^ and gradually increased to slightly above 30 MtCO_2_eq yr⁻^1^, eventually surpassing NPP post-2050.

The NEP, calculated as NPP minus *R*_h_, was initially positive at approximately 13 MtCO_2_eq yr⁻^1^, indicating the forest acted as a carbon sink (Fig. 3 (b)). However, NEP dropped to around 4 MtCO_2_eq yr⁻^1^ after the first outbreak and turned negative following the second outbreak period (2042–2049), signaling a transition to a carbon source. Thus, the NBP— which accounts for both ecosystem balance and disturbance-driven losses— mirrored this trend and reached as low as –2 MtCO_2_eq yr⁻^1^ by the end of the simulation. Overall, consecutive SBW outbreaks (2010–2017; 2042–2049; 2074—2081) accelerated the forest's transition from a carbon sink to a carbon source.

Under RCP2.6 and RCP4.5, the decline in NPP was less severe and consistently exceeded *R*_h_, allowing the ecosystem to maintain its role as a net carbon sink throughout most of the simulation. Toward the end of the period, both RCP2.6 and RCP4.5 showed improved carbon sequestration capacity, reinforcing ecosystem recovery. In contrast, under RCP8.5, NPP declined more substantially, reaching about 29 MtCO_2_eq yr⁻^1^ by 2110. *R*_h_ increased throughout the century, peaking at nearly 34 MtCO_2_eq yr⁻^1^, which led to persistent negative NEP and a high reduction of NBP to –19 MtCO_2_eq yr⁻^1^ in 2070—the most severe carbon loss across all scenarios.

## Effect of salvage harvesting on carbon dynamics

### Effect on carbon pools

The implementation of additional salvage biomass scenarios after spruce budworm (SBW) outbreaks had a modest effect on the size of the DOM carbon pool (Fig. 3 (c)). Across all RCP climate scenarios, the changes in total DOM storage under salvage scenarios S1, S2, and S3 reached approximately 0.75, 1.25, and 3 MtCO_2_eq yr⁻1, respectively, relative to the reference scenario (S0) (Fig. 3 (c)). The magnitude of DOM reduction scaled proportionally with salvage intensity but remained small relative to total ecosystem carbon storage.

### Effect on carbon fluxes

The implementation of salvage biomass harvesting (scenarios S1 to S3) led to a reduction in *R*_h_ across all climate scenarios, with an average Δ of approximately –0.01 MtCO_2_eq yr⁻^1^ in S1, –0.014 MtCO_2_eq yr⁻^1^ in S2, and –0.025 MtCO_2_eq yr⁻^1^ in S3 compared to the S0 (Fig. 3 (c)). The observed decline in *R*_h_ is attributed to the removal of deadwood through salvage harvesting, which limits microbial decomposition and subsequently reduces carbon emissions from the DOM pool. This reduction in decomposition-related emissions was particularly pronounced under RCP8.5, where the maximum decrease in *R*_h_ (ΔRh) reached approximately − 0.05 MtCO₂eq yr⁻1 by the end of the simulation. Under baseline climate, the maximum reduction was − 0.035 MtCO₂eq yr⁻1, indicating that warming amplified the benefits of deadwood removal.

As a result, NEP improved slightly under all salvage scenarios (Figure A7). Despite these positive effects on *R*_h_ and NEP, the impact on net biome production (NBP) remained minimal, with delta average generally below –0.05 MtCO_2_eq yr⁻^1^, indicating that salvage harvesting had a limited influence on long-term carbon sequestration at the landscape scale. The NBP delta was negative during outbreak periods and positive between outbreaks, while salvage harvesting reduced decomposition emissions by up to 0.05 MtCO₂eq yr⁻1, with larger reductions under warmer climate scenarios (Figure A8). In contrast, avoided fire emissions were negligible (< 0.001 MtCO₂eq yr⁻1) across all climate and salvage scenarios (Figure A8).

### Transport and other operations carbon emissions

**Fig. 4:**
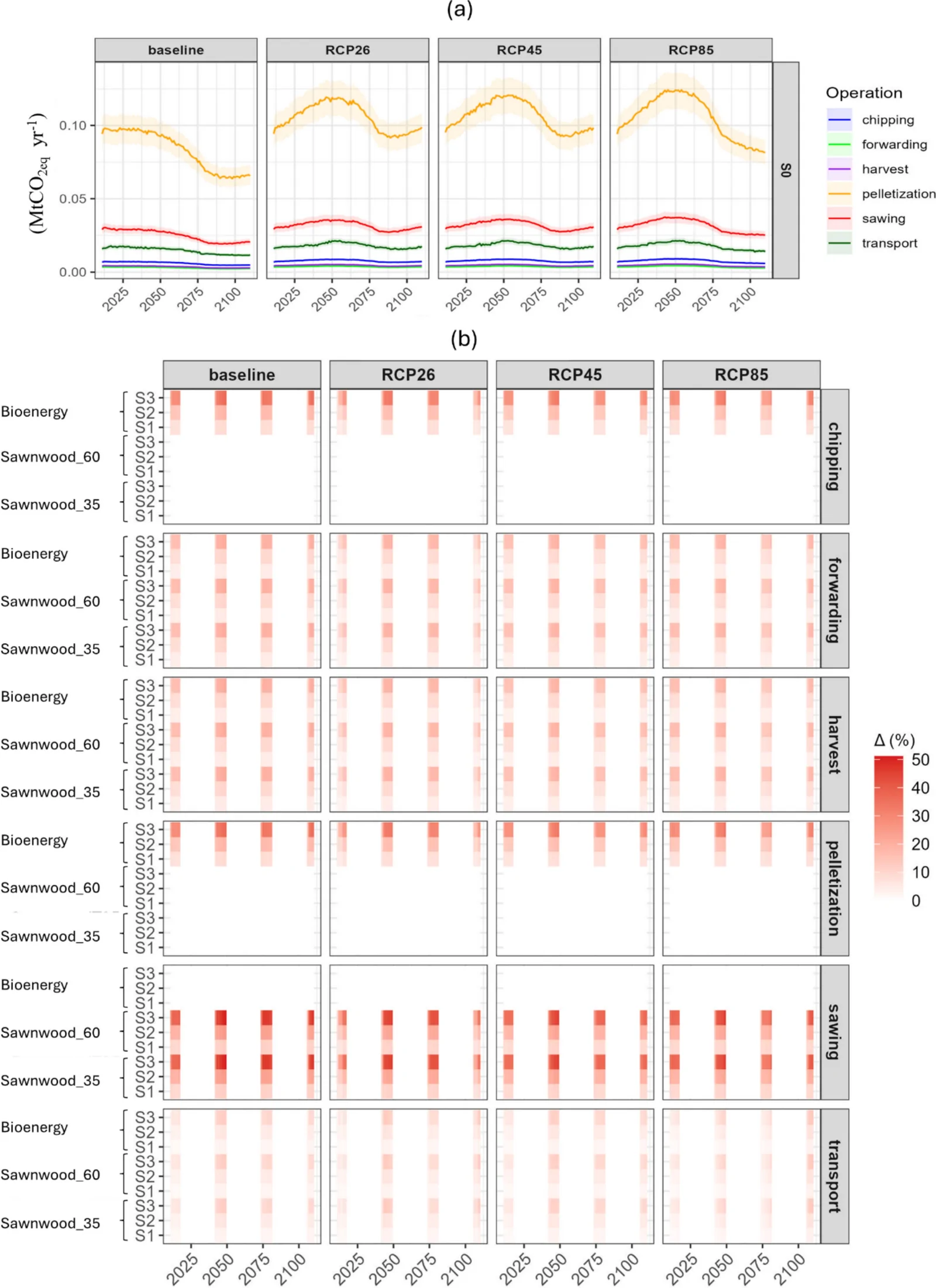
Operational emissions (Mt CO₂eq yr⁻^1^) from the forest-biomass supply chain. **a** Under reference management (S0), pelletization dominates emissions, followed by sawing; field operations (harvesting, forwarding, transport) remain minor contributors across all Representative Concentration Pathways (RCP). **b** Heat-map showing percentage change (Δ%) relative to S0 for salvage scenarios S1–S3 under three wood-use pathways: salvage increases mill-stage emissions by 10–50%, with pelletization most responsive when biomass is directed to bioenergy

Our results shown that carbon emissions from transport and processing operations in the boreal forest supply chain were relatively modest compared to the ecosystem and HWP. However, these emissions still exhibit sensitivity to both climate and management scenarios (Fig. 4 (a)). Under baseline climate, transport averages about 0.012 Mt CO₂eq yr⁻^1^. As climate forcing intensifies, transport rises ~ 10% (RCP2.6), ~ 22% (RCP4.5), and ~ 33% (RCP8.5) versus baseline. During the SBW outbreak years, adding salvage biomass increases transport by roughly ~ 3% (S1, 1 000 ha), ~ 7% (S2, 2 000 ha), and ~ 12% (S3, 4 000 ha) relative to S0 (Fig. 4 (b)).

Under S0 (no additional salvage), mill-stage emissions were dominated by pelletization, averaging ~ 0.09–0.11 Mt CO₂eq yr⁻^1^ across climate pathways, followed by sawing (~ 0.035–0.05 Mt CO₂eq yr⁻^1^) and transport (~ 0.012 Mt CO₂eq yr⁻^1^), whereas chipping, harvesting, and forwarding each remained < 0.02 Mt CO₂eq yr⁻^1^. When salvaged biomass was directed to bioenergy, pelletization increased by ≈ + 15% (S1), + 25–30% (S2), and + 35–45% (S3) versus S0. When salvaged biomass was routed to sawnwood, sawing increased by + 10–20% (S1), + 20–35% (S2), and + 35–50% (S3) for the 35-year half-life stream, and by + 15–25% (S1), + 25–40% (S2), and + 40–50% (S3) for the 60-year half-life stream. The emissions profile overall reveals that industrial processing dominates the carbon footprint, while field operations–harvesting, forwarding and transport–have a limited effect (~ + 3–15% relative to S0). Climate change pathways further amplify this pattern, especially under RCP4.5 and RCP8.5 (Fig. 4 (b)).

### Carbon sequestration by harvested wood products

**Fig. 5:**
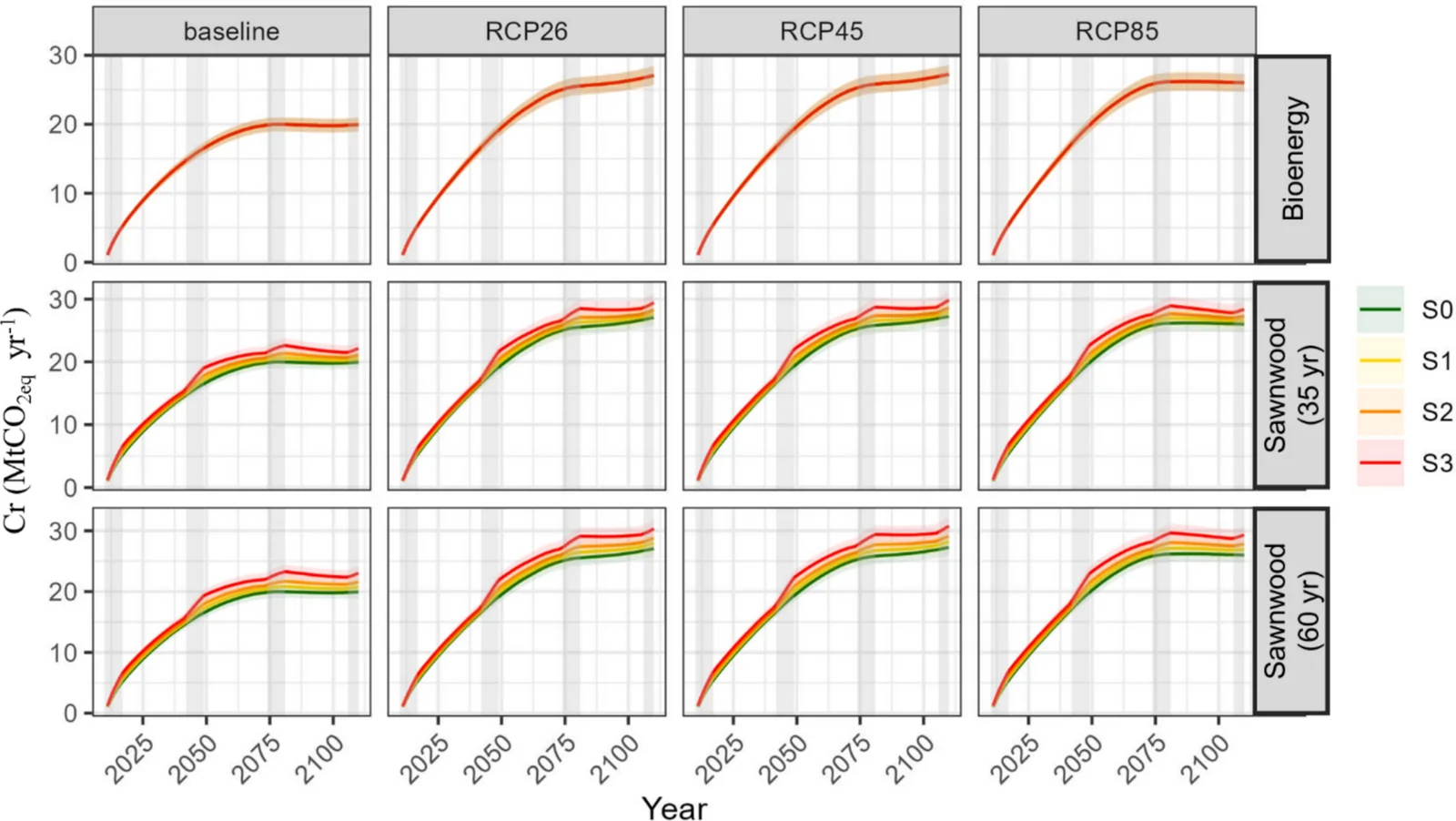
Carbon retention in harvested wood products (HWP) increases with salvage intensity and product longevity. Total carbon remaining (Cr; MtCO_2_eq) after accounting for decomposition and combustion emissions is shown for salvage scenarios S0–S3 under three wood-use pathways. Directing salvaged biomass to sawnwood (especially 60-year half-life) substantially increases HWP storage, whereas bioenergy yields no additional retention. Vertical gray bands indicate spruce budworm (SBW) outbreak periods. See Figure A10for differences (Δ) relative to S0

Under S0, annual carbon transfers to harvested wood products (HWP) increase rapidly, reaching approximately 20 MtCO₂eq yr⁻1 under baseline climate conditions and exceeding 26 MtCO_2_eq yr⁻1 across all RCP climate pathways (Fig. 5). Sawnwood accounts for roughly 90% of the remaining carbon in the HWP pool, with the remaining 10% stored in pulp and paper (Figure A9). Annual emissions from HWPs are relatively small compared to the inflow, reaching approximately 0.2 MtCO₂eq yr⁻1 from bioenergy, 1 MtCO₂eq yr⁻1 from sawnwood (35-year half-life), and 1.5 MtCO₂eq yr⁻1 from pulp and paper (Figure A9).

When additional salvaged biomass during the post-outbreak is allocated to bioenergy, all harvested biomass is immediately emitted to the atmosphere. The resulting additional emissions from bioenergy scale with salvage intensity, reaching more than 0.05, 0.10, and 0.20 MtCO₂eq yr⁻1 for S1, S2, and S3, respectively (Figure A10). In contrast, allocating salvaged biomass to long-lived wood products substantially increases carbon storage in HWPs. For sawnwood with a 35-year half-life, the additional remaining carbon reaches approximately 0.6, 1.1, and 2.5 MtCO₂eq yr⁻^1 ^for S1, S2, and S3, respectively, by the end of the simulation under current climate conditions. Incremental emissions from sawnwood with a 35-year half-life also rise with salvage intensity, reaching approximately 0.01, 0.02, and 0.04 MtCO₂eq yr⁻1 for S1, S2, and S3, respectively (Figure A10). When the salvaged biomass is instead allocated to sawnwood with a 60-year half-life, the increase in remaining carbon is approximately doubled compared to the 35-year case, while emissions from decomposition are proportionally lower. Across product pathways, warmer climate scenarios increased HWP carbon inputs by enhancing biomass mobilization, leading to higher cumulative carbon retention when salvaged biomass was allocated to long-lived products (Fig. 5).

### Forest net sector production

**Fig. 6:**
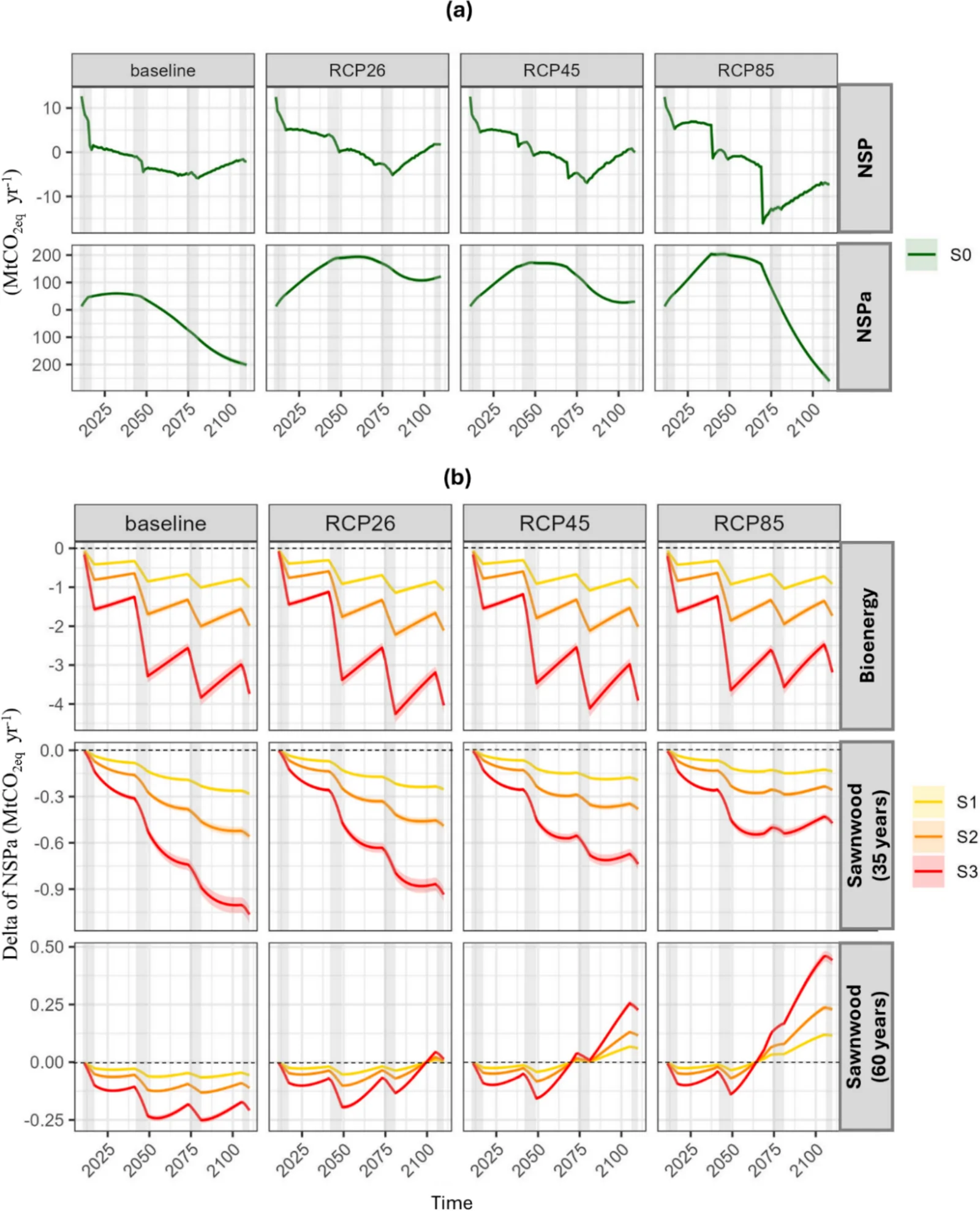
Temporal trajectories of forest sector carbon under four climate pathways. Net sector production (NSP) trajectories under four climate pathways (baseline, RCP2.6, RCP4.5, RCP8.5). **a** Annual NSP (MtCO₂eq yr^−1^) and cumulative NSP (NSPa; MtCO_2_eq) for reference management (S0); NSPa becomes negative by mid-century under baseline and RCP8.5, indicating net carbon loss from the forest sector. **b** Incremental effect of salvage (ΔNSPa) for three intensities (S1 = 1,000 ha; S2 = 2,000 ha; S3 = 4,000 ha) and three wood-use pathways: bioenergy produces persistent penalties; short-lived sawnwood (35—yr half-life) accumulates decomposition losses; only long-lived sawnwood (60—yr half-life) yields net sector benefits, outperforming S0 under RCP4.5–RCP8.5 by late century. The delta of NSP compared to other scenarios is shown in Figure A12

Under the reference management scenario (S0), NSP declined sharply during spruce budworm (SBW) outbreaks, with the strongest reduction occurring during the first outbreak (Fig. 6). Under the baseline climate, NSP decreased from approximately 10–12 MtCO₂eq yr⁻1 in the early 2020s to near zero by ~ 2030, became negative during subsequent outbreaks (− 3 to − 5 MtCO₂eq yr⁻1 in the 2060s–2070s), and partially recovered by the end of the simulation. Under moderate climate forcing (RCP2.6 and RCP4.5), NSP was consistently higher than under the baseline climate, remaining positive until mid-century and peaking near ~ 30 MtCO₂eq yr⁻1 (compared to ~ 20 MtCO₂eq yr⁻1 under baseline). After 2070, NSP declined under both scenarios, reaching approximately − 5 MtCO₂eq yr⁻1 under RCP2.6 and − 6 to − 7 MtCO₂eq yr⁻1 under RCP4.5 before partially recovering by the late century (Fig. 6). In contrast, under RCP8.5, NSP exhibited the strongest deterioration: following a mid-century increase, it declined sharply after ~ 2070, reaching − 8 to − 10 MtCO₂eq yr⁻1 and remaining negative through 2110, reflecting increased *R*_h_ reduced forest productivity (Fig. 3).

On the other hand, cumulative NSP— which indicated the total net carbon gain or loss — increased slowly to about + 40–60 MtCO₂eq yr⁻1 by mid-century under the baseline climate (Fig. 6), then declined, crossed zero around 2065–2070, and reached − 200 by 2110. In RCP2.6, cumulative NSP increased rapidly to about + 200 near 2065–2070, then reduced to ~  + 100 by 2110. RCP4.5 peaked around + 160–180 near 2060, then declined to ~  + 50 by 2120. In RCP8.5, it exceeded + 200 by mid-century but then fell sharply, crossed zero before 2090, and ended near − 240 by 2110, indicating a strong net loss under the extreme climate change pathway.

The effect of salvage biomass after SBW outbreaks depended strongly on the utilization pathway. When salvaged biomass was directed to bioenergy, emissions spiked immediately during each outbreak period, and the change in cumulative NSP relative to S0 (ΔNSPa) remained negative throughout the century; this penalty increased with salvage intensity (S1 < S2 < S3) (Fig. 6). Emission partitioning shows that in the bioenergy pathway (Figure A11), most of this penalty originated from the HWP component due to immediate combustion emissions, with negligible ecosystem emission savings. Using salvaged biomass for sawnwood with a 35-year half-life reduced combustion emissions compared to bioenergy, but still resulted in a larger long-term ΔNSPa decline. In contrast, directing salvaged biomass to sawnwood with a 60-year half-life produced the lowest total emissions: although ΔNSPa was negative early in the simulation, it later stabilized and partially recovered—especially under RCP4.5 and RCP8.5—approaching zero or becoming slightly positive by late century. Under baseline and RCP2.6, ΔNSPa remained negative for all uses; under RCP4.5 and RCP8.5, it offset much of the penalty, and in some cases yielded a net benefit when salvage was routed to 60-year sawnwood.

## Discussion

In the boreal forest of eastern Canada, particularly in the Côte-Nord region, disturbances such as fire, insect outbreaks, and logging significantly influence the spatial patterns of forest age and species composition, altering the dynamics of carbon storage and flux. Using the LANDIS-II model, this study projects the impacts of natural disturbances, with a focus on spruce budworm outbreaks, and evaluates the effects of salvage biomass strategies on carbon dynamics. Our findings reveal that: *i*) Climate change is the predominant driver of carbon dynamics in this region, affecting both the rate of carbon sequestration and emissions; *ii*) Salvage biomass strategies can mitigate part of the carbon loss from post-outbreak decomposition and increase the harvested wood product (HWP) contribution to total carbon retention, but the magnitude and direction of the impact depends strongly on the wood-use pathway and product longevity.

### Climate change effects on carbon dynamics at ecosystem scale

Under current climate conditions, simulated NPP at the beginning of the analysis period (2010) ranged from 2–3 tC ha⁻^1^ yr⁻^1^, closely matching values reported in regional forest inventories (Fig. 3a). Initial AGB averaged ~ 25 tC ha⁻^1^, consistent with estimates of 10–35 tC ha⁻^1^ reported for the Côte-Nord region (Boudreau et al. 2008; Duchesne et al. 2016). These comparatively low productivity levels reflect the edaphic and climatic constraints characteristic of this northern boreal environment, including cold temperatures and nutrient limitations (Jobidon et al. 2015), and help explain why NPP is lower than in southern Québec forests (Liu et al. 2002). Also, simulated soil carbon stocks averaged ~ 80 tC ha⁻^1^ (Fig. 3a), falling well within the regional range of 60–131 tC ha⁻^1^ reported by (Paré et al. 2011a). Consistent with boreal forest carbon budgets, NPP and Rh dominate annual carbon fluxes, with approximately 10% of NPP contributing to NEP under current climate conditions (Kurz et al. 2013).

Climate change strongly influences forest productivity, composition, and carbon dynamics. Warming initially enhances NPP and biomass by extending the growing season and reducing cold-related growth limitations (Boulanger et al. 2017; D’Orangeville et al. 2018). These positive effects dominate the early to mid-twenty-first century, as milder winters and longer growing seasons stimulate growth across species (Ameray et al. 2023a). However, under the high-emissions scenario (RCP8.5), productivity declines sharply after ~ 2070, particularly in coniferous species such as black spruce. Increased drought stress, tree mortality, and fire activity reduce NPP and biomass accumulation, shifting forests from a carbon sink to a net carbon source (Gauthier et al. 2015; Boulanger et al. 2017; Boulanger and Puigdevall 2021; Ameray et al. 2024). Simultaneously, higher disturbance rates alter forest age structure, reducing the prevalence of older stands and limiting long-term carbon sequestration as mature conifer forests are progressively replaced by younger, more mixed stands (Bergeron et al. 2014; Liang et al. 2018).

The dynamics of DOM carbon storage are closely linked to AGB across different species and climate scenarios. Under baseline conditions, increases in AGB across all species promote continued accumulation of DOM. Under climate change scenarios, DOM stocks generally remain stable or increase, except under RCP8.5 after ~ 2070. This relative stability reflects the dominance of coniferous species, whose litter and woody debris decompose more slowly than those of broadleaf species(Laganière et al. 2015; Hararuk et al. 2017; Hüblová and Frouz 2021). In contrast, the decline in DOM under RCP8.5 is driven by higher temperatures that accelerate decomposition, consistent with previous findings (Hüblová and Frouz 2021; Ameray et al. 2024). Although warming enhances decay rates under all RCPs, only under RCP8.5 does a concurrent decrease in NPP lead to an imbalance between carbon inputs and losses, resulting in net DOM reduction. Under RCP2.6 and RCP4.5, increased productivity offsets enhanced decomposition, sustaining or slightly increasing DOM stocks.

### Spruce budworm effect on forest carbon dynamics

Spruce budworm is a significant ecological driver in the boreal forest, profoundly impacting composition, age structure, and carbon dynamics (Dymond et al. 2010; Navarro et al. 2018; Pureswaran et al. 2018, 2019). Using LANDIS-II, our simulations realistically reproduce regional forest responses to SBW outbreaks, with mortality patterns closely matching historical observations from 1967 to 1992. Model calibration focused on tree mortality rather than defoliation, as aerial defoliation surveys may overestimate SBW impacts by conflating them with drought stress or late frost damage (Senf et al. 2017). Previous work has demonstrated that SBW vulnerability is strongly mediated by stand composition and age, with mixed stands sustaining lower damage than pure host-species stands (Sturtevant et al. 2004). Consistent with prior work in the region, mixtures of black spruce and balsam fir contribute to reduced SBW-induced mortality under climate change, particularly under RCP4.5 and RCP8.5 (Ameray et al. 2024). Nevertheless, similar to Dymond et al. (2010), our results indicate substantial ecosystem carbon losses from SBW outbreaks, reaching 2–15% over a 14-year outbreak period.

## Salvage biomass effects on sector-level carbon dynamics

### Ecological mechanisms

**Fig. 7:**
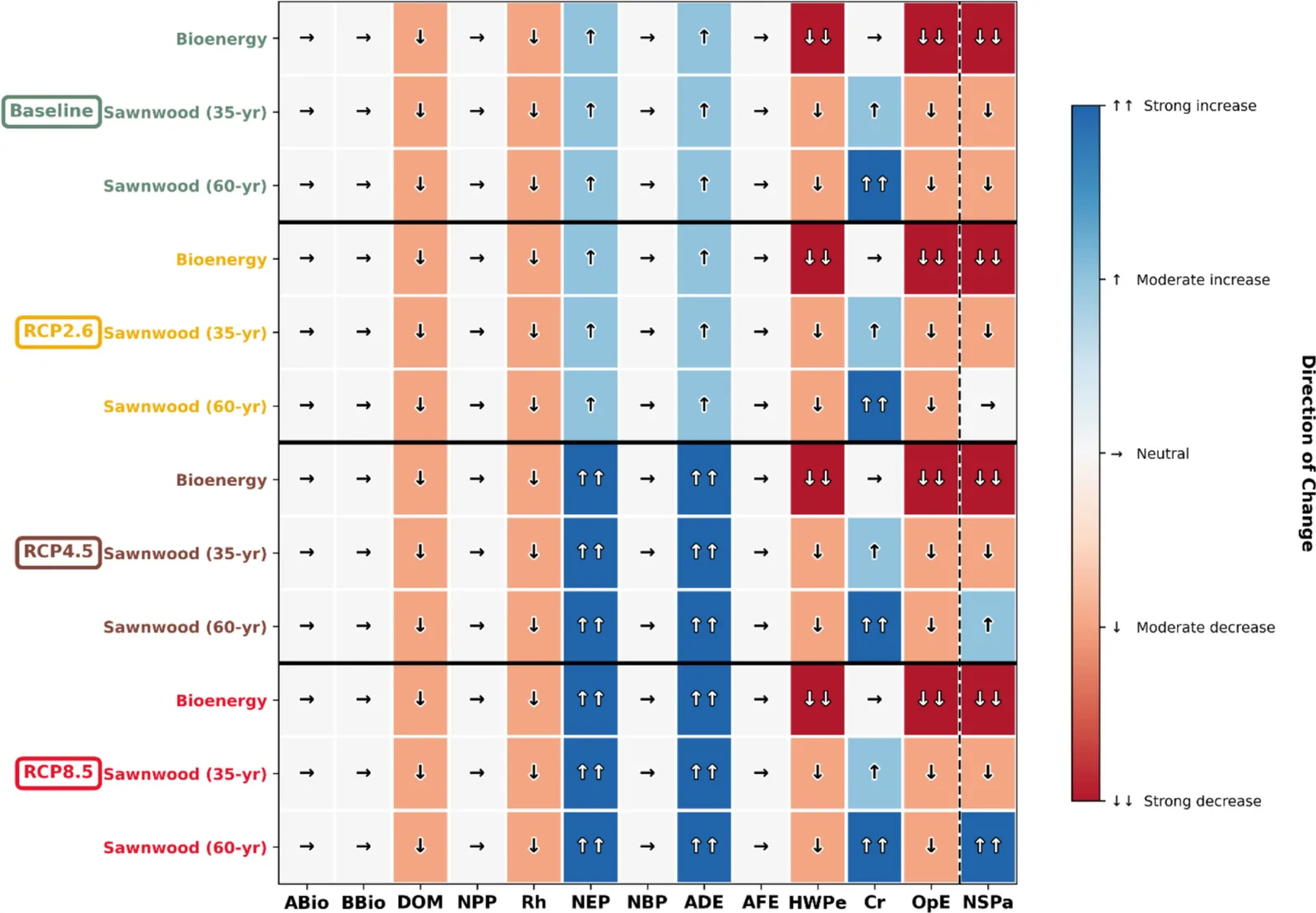
Late-century synthesis of salvage-biomass effects across climate pathways and product uses. Variables shown: ecosystem pools (ABio = aboveground biomass; BBio = belowground biomass; *DOM*   dead organic matter), fluxes (*NPP* net primary productivity; *R*_h_ heterotrophic respiration; *NEP* net ecosystem productivity; *NBP* net biome productivity), avoided emissions from decomposition and fire (ADE, AFE), HWP emissions (HWPe), carbon remaining in HWP (Cr), operational emissions (OpE), and cumulative net sector production (NSPa). Net sector benefits (positive NSPa) occur only when salvaged biomass is directed to long-lived sawnwood (60—yr half-life) under RCP4.5 and RCP8.5, where accelerated decomposition increases the value of removing deadwood

Salvage harvesting influences ecosystem carbon balance primarily by reducing *R*_h_. SBW–induced mortality transfers biomass to deadwood pools (snags and coarse woody debris), where it decomposes. Decomposition rates are strongly temperature dependent, following a Q₁₀ relationship in which rates approximately double for each 10 °C increase (Hararuk et al. 2017). By removing a portion of this dead biomass before decay, salvage harvesting limits the substrate available for *R*_h_ and thereby reduces ecosystem carbon emissions. Salvage effects, therefore, appear higher in RCP4.5 and RCP8.5 pathways than in baseline and even RCP2.6 (Fig. 7). Under warmer pathways, accelerated decay causes deadwood left on site to release carbon more rapidly, increasing the relative benefit of removing SBW-killed stems. As a result, avoided *R*_h_ fluxes from salvage harvesting are proportionally larger under RCP4.5–RCP8.5, where mean annual temperatures rise by 5–7.5 °C by 2100, compared with the ~ 3 °C increase projected under RCP2.6.

Salvage harvesting can also reduce the probability of additional carbon losses from fire or insect outbreaks (Mansuy et al. 2018; Gunn et al. 2020; Gouge et al. 2021). Our results show that avoided emissions from salvage harvesting are primarily attributable to reduced decomposition rather than fire prevention (Figure A8). This finding aligns with Gouge et al. (2021) and Gunn et al. (2020), who similarly concluded that the carbon balance benefits of salvage stem largely from avoided decomposition. In our study, fire-related emissions displayed minimal differences across salvage scenarios due to their stochastic nature—ignition patterns vary independently from outbreak locations.

### Forest-sector implications: HWP outcomes and net sector production

Overall, our results demonstrate that salvage biomass utilization strategies strongly depend on the long-term carbon dynamics of HWPs. The greatest gains in carbon sequestration in wood product pool are achieved when salvaged biomass is directed to long-lived products with extended half-lives, whereas use for bioenergy yields only short-term emission pulses without contributing to long-term storage (Figure A10). When these HWPs displace carbon-intensive materials such as steel or concrete, additional fossil fuel and process emissions are avoided, further improving the carbon balance (Howard et al. 2021; Cardinal et al. 2024), though such substitution effects were not evaluated in this study.

In our study, the sector-level benefit of salvage harvesting depends on both climate change and product allocation. Directing salvaged biomass to long-lived sawnwood can improve the forest-sector carbon budget under RCP4.5 and RCP8.5 (Fig. 7), where warming and more frequent disturbances accelerate on-site decomposition and increase the value of transferring carbon into longer-lived HWP pools (Kurz et al. 2016; Gregor et al. 2024). By contrast, under the baseline climate and RCP2.6, slower decay and limited disturbance allow carbon to persist longer in deadwood and soils, which reduces within-boundary gains from additional post-outbreak extraction (Kurz et al. 2016; Gregor et al. 2024). Emission partitioning (Figure A11) clarifies these differences across wood-use pathways: bioenergy generates a persistent penalty because emissions are dominated by immediate combustion with negligible ecosystem savings, while 35-year sawnwood reduces combustion but still accumulates product decomposition emissions that outweigh small ecosystem benefits. Only 60-year sawnwood yields the lowest total emissions and the strongest recovery in ΔNSPa—negative early but stabilizing and approaching zero or slightly positive by late century, especially under RCP4.5–RCP8.5 (Figure A11).

Our results further indicate that the modest magnitude of sector-level carbon balance improvements observed across scenarios is largely attributable to the relatively small salvage areas represented in the simulations. Although increasing the targeted salvage area would proportionally enhance avoided decomposition emissions and long-lived HWP carbon storage—thereby improving NEP, NBP, and NSP—such scaling is not unconstrained. In practice, the extent to which salvage harvesting can be expanded is limited by operational costs, industrial processing capacity, transportation logistics, and, critically, the spatial accessibility and concentration of spruce budworm–affected stands. As a result, the carbon benefits identified here should be interpreted as conditional on both disturbance extent and supply-chain feasibility.

Salvage biomass operations increase emissions associated with the biomass supply chain. Our analysis considered emissions from critical stages—harvesting, forwarding, transport, sawing, chipping, and pelletization—across different climate and salvage scenarios. Increasing salvage intensity led to a 7–20% rise in transport-related emissions, directly linked to higher biomass volumes and longer haul distances. This pattern is consistent with Giasson et al. (2023), who show that transport emissions are non-negligible but remain small relative to overall forest-sector carbon dynamics. Among supply-chain components, pelletization was consistently the most carbon-intensive process, with emissions reaching up to 0.15 MtCO₂eq yr⁻1 under S3, reflecting the high energy demand for drying and densification—especially in cold climates (Laganière et al. 2017; Mansuy et al. 2018). Sawing ranked second in (~ 0.05 MtCO₂eq yr⁻1), driven by electricity-intensive milling, cutting, and debarking, consistent with previous assessments of sawnwood production emissions (Paré et al. 2011b; Giasson et al. 2023). In contrast, harvesting and forwarding remained minor contributors (< 0.01 MtCO₂eq yr⁻1) across all scenarios. Overall, mill processes dominate the supply-chain footprint, while field operations remain minor, consistent with prior LCAs (Giasson et al. 2023; Lamers et al. 2014).

In addition to operational emissions, salvage harvesting presents potential ecological risks. The large-scale removal of biomass, particularly coarse woody debris, may reduce nutrient availability and alter microsite conditions, thereby impeding regeneration and reducing future productivity (Garrett et al. 2021; Gouge et al. 2021). However, in our simulations a large fraction of SBW-killed biomass (e.g., branches, foliage, and sub-merchantable stems) remained on site and continued to contribute to DOM pool, which likely limited both the ecosystem-scale carbon response and potential nutrient depletion risks. We also simulated best-management practices (e.g., retaining 10% old-growth and maintaining natural regeneration) to reduce these risks. Nevertheless, the viability and sustainability of salvage biomass as a carbon management strategy remain contingent on context-specific implementation and the actual efficacy of these practices in the long-term. Future work should aim to integrate field data on regeneration success, nutrient cycling, and decay rates of retained biomass, especially if salvage harvesting is to be scaled within broader climate policy frameworks.

### Study limitations and improvements

While this study advances the understanding of how salvage biomass affects forest-sector carbon dynamics, several limitations remain that warrant consideration.

First, although we modeled carbon dynamics across the full harvested wood product (HWP) supply chain—including harvesting, forwarding, sawing, chipping, pelletization, and transport—our approach excluded substitution effects. In line with the IPCC Tier 2 methodology, we conservatively assumed that bioenergy use results in immediate carbon emissions and did not account for the potential displacement of fossil fuel-based energy. This likely underestimates the net climate benefits of salvage biomass. Several studies have demonstrated that incorporating substitution can significantly increase net GHG reductions—by up to 40 MtCO₂eq yr⁻1 in Canada by 2050, when combining HWP storage with fossil fuel displacement (Cherubini et al. 2009; Smyth et al. 2014, 2017; Laganière et al. 2017). Moreover, Smyth et al. (2017) emphasize that material substitution by long-lived wood products (e.g., lumber, engineered panels) can also offset emissions from carbon-intensive building materials. As such, our exclusion of substitution effects likely leads to a conservative estimate of the full mitigation potential of salvage biomass strategies.

Second, the salvage operations applied in our simulations focused exclusively on dead snag stem harvesting and did not capture the full operational complexity of salvage logging. In practice, salvage operations often include partially defoliated or vulnerable living trees to prevent subsequent degradation or mortality. This nuance, while beyond the scope of our current work, may influence the actual volume of biomass recovered and the carbon outcomes of real-world operations.

Third, the SBW simulation model is based on Biological Disturbance Agent extension mortality functions, which estimate the extent of an outbreak in relation to the proportion of different host species, age structure of hosts, and neighborhood effects (Sturtevant et al. 2004). However, in nature, SBW outbreaks are influenced by a range of other processes not included in the model, such as natural enemies of SBW, including parasitoids, predators, and entomopathogens; climatic effects on insect phenology and survival; and complex interactions with forest composition, which may affect outbreak intensity and duration (Pureswaran et al. 2019). These omitted controls could alter outbreak intensity and duration nder novel climates. Although our BDA parameterization is based on historical mortality records from 1967 to 1992, the absence of these biotic interactions represents a simplification that may affect projected outbreak dynamics, particularly under novel climate conditions where such interactions could shift substantially.

Fourth, our modeling framework likely underestimates fire-salvage interactions. In the LANDIS-II Base Fire extension, fire ignition is stochastic and spatially independent of outbreak locations, meaning that the probability of fire occurring in SBW-affected stands is not explicitly elevated despite increased fuel loads from snag accumulation. In reality, stands with high snag densities following insect outbreaks may experience elevated fire risk due to changes in fuel structure, moisture content, and continuity (James et al. 2017). Consequently, our estimates of avoided fire emissions from salvage harvesting (Figure A8) may be conservative, and the carbon balance benefits of salvage could be larger than simulated if fire-salvage interactions were more realistically represented.

Finally, this study did not address biodiversity impacts or ecological feedbacks associated with salvage harvesting. Deadwood provides critical habitat for cavity-nesting birds and saproxylic insects (Müller et al. 2020). Future work should integrate biodiversity indicators, and disturbance feedback mechanisms to provide a more comprehensive assessment of salvage sustainability.

Although the modeling framework relies on validated components, direct validation of simulated carbon pools and fluxes against independent observations remains limited. Initial aboveground biomass was validated against the 4th National Forest Inventory, showing no regional bias but spatially heterogeneous errors, particularly in northern land types where paludification and organic soils constrain growth processes not represented in LANDIS-II. Despite these limitations, confidence in the results is supported by the use of the CBM-CFS3–based ForCS module (IPCC Tier 3 compliant), prior calibration of PnET-II with yield curves and remote sensing data, consistency of simulated NPP, biomass, and soil carbon with regional estimates, and calibration of SBW mortality using historical aerial surveys (1967–1992). Future work should prioritize validation using eddy-covariance data and repeated post-outbreak inventories.

Our approach represents a significant methodological improvement by integrating spatially explicit forest dynamics with full-supply-chain carbon accounting. It demonstrates the feasibility of using forest landscape models, such as LANDIS-II, to assess the sector-wide mitigation potential of salvage biomass strategies under varying climate scenarios. Future work could further enhance this framework by combining empirical decay models, operational constraints, and substitution dynamics to provide more robust estimates of carbon outcomes across the forest value chain.

## Conclusion

We quantified the forest-sector carbon consequences of salvage biomass harvesting following spruce budworm outbreaks in eastern Canadian boreal forests within a system boundary excluding substitution effects. Overall, salvage harvesting can improve sector-level carbon balances, but only under specific climate and product-use conditions. Under higher warming scenarios (RCP4.5 and RCP8.5) with long-lived products, salvage operations approach carbon–neutral or slightly positive outcomes by late century; under baseline and RCP2.6, salvage does not yield net carbon benefits within the modeled boundary. Using spatially explicit modeling with full supply-chain carbon accounting, we found that directing salvaged biomass into long-lived harvested wood products (HWPs)—especially sawnwood with extended half-lives—enhances long-term HWP storage and partially offsets on-site decomposition losses, with the strongest benefits under RCP4.5 and RCP8.5. Although supply-chain emissions rise with salvage intensity—driven mainly by pelletization and sawing—they remain small relative to the total carbon budget.

The within-boundary outcome is highly sensitive to allocation pathways: without substitution, bioenergy use creates a carbon penalty because emissions occur immediately at combustion, whereas long-lived products retain carbon over decades when their half-lives are long relative to DOM persistence. Under baseline and low-warming scenarios, slower decay and lower disturbance limit the value of additional post-outbreak extraction, and any broader mitigation would depend on substitution offsetting added emissions from bioenergy or shorter-lived HWPs. Under higher warming, faster decay and greater disturbance increase the potential value of removing a fraction of SBW-killed stems, but our modeling suggests net benefits emerge mainly under strong warming and when additional biomass is stored in very long-lived HWPs. Future work should incorporate empirically constrained substitution, operational variability, and post-harvest ecosystem responses to better capture economy-wide benefits and trade-offs of salvage strategies at landscape scales.

## Supplementary Information

Below is the link to the electronic supplementary material.Supplementary file1 (DOCX 7758 KB)

## Data Availability

All Data and scripts used for the simulation setup are available on GitHub. (https://github.com/Ameray/Landis-II-SalvageBiomass/tree/main)

## Abbreviations

ABio: Above biomass
BBio: Below biomass
DOM: Dead organic matter
HWP: Harvested wood products
Pest: Establishment probability
ForCS: Forest Carbon Succession
*R*_h_: Heterotrophic respiration
LCA: Life cycle assessment
MaxAGB: Maximum aboveground biomass
MaxANPP: Maximum aboveground net primary production
MtCO₂eq: Megatons of CO_2_ equivalent
NBP: Net biome productivity
NEP: Net ecosystem productivity
NPP: Net primary productivity
NSP: Net sector production
NSPa: Cumulative net sector production
RCP: Representative Concentration Pathway
SBW: Spruce budworm
