# Supplementary material for: Landscape carbon trajectories after spruce budworm outbreaks in Canada’s Eastern boreal forest: effects of salvage intensity and wood-use pathways
Source: Landsc Ecol. 2026 Jun 6;41(8):131. doi: 10.1007/s10980-026-02376-1 (PMC13421357; doi:10.1007/s10980-026-02376-1)

**Supplementary materials**

**Table A.1.** Decay rates used in ForCS. We used Hararuk et al. (2017) proposed values by group (softwood vs hardwood) rather than CBM-CFS3 default values. Delta (%) reflects the difference between hardwood and softwood decays.

| **pool** | **Description** | **Default value CBM-CFS3** | **Hararuk et al (2017) values** | | **Delta (%)** |
| --- | --- | --- | --- | --- | --- |
|  |  |  | **Softwood** | **Hardwood** |  |
| "Very Fast Aboveground" | Foliar litter plus dead fine roots, approximately <5mm diameter | 0.355 | 0.345 | 0.345 | 0 |
| "Very Fast Belowground" |  | 0.5 | 0.349 | 0.349 | 0 |
| "Fast Aboveground" | Fine and small woody debris plus dead coarse roots in the forest floor, approximately ≥5 and <75mm diameter. | 0.1435 | 0.16 | 0.23 | 44 |
| "Fast Belowground" |  | 0.1435 | 0.16 | 0.23 | 44 |
| "Medium" | Coarse woody debris on the ground. | 0.0374 | 0.05 | 0.11 | 120 |
| "Slow Aboveground" | F, H and O horizons. | 0.015 | 0.022 | 0.022 | 0 |
| "Slow Belowground" | Humified organic matter in the mineral soil. | 0.0033 | 0.004 | 0.004 | 0 |
| "Stem Snag" | Dead standing stemwood of merchantable size | 0.0187 | 0.014 | 0.054 | 286 |
| "Other Snag" | Dead branches, stumps and small trees | 0.07175 | 0.047 | 0.073 | 55 |

**Table A.2**. Example of Forest Carbon Succession (ForCS) extension parameters for ecoregion eco1SALO under baseline climate conditions. *Pest*: Species annual establishment probability (prob.yr-1); probability that seeds of a species will establish on suitable sites given adequate light and seed availability. MaxANPP: Maximum aboveground net primary productivity (g/m²/yr); upper limit of annual carbon accumulation in aboveground biomass under optimal conditions. MaxAGB: Maximum aboveground biomass (g/m²); carrying capacity representing the upper limit of biomass a cohort can attain at maturity. Complete parameter files for all ecoregions are available on GitHub.

| **Species** | **Parameter** | **Value** |
| --- | --- | --- |
| Yellow Birch | Pest | 0.00856 |
| Paper Birch | Pest | 0.00855 |
| White Spruce | Pest | 0.00259 |
| Black Spruce | Pest | 0.10923 |
| Tamarack Tree | Pest | 0.11501 |
| Jack Pine | Pest | 0.006 |
| Trembling Aspen | Pest | 0.00857 |
| Balsam Fir | Pest | 0.04191 |
| Yellow Birch | MaxANPP | 943.8 |
| Paper Birch | MaxANPP | 740.6 |
| White Spruce | MaxANPP | 810.7 |
| Black Spruce | MaxANPP | 836.2 |
| Tamarack Tree | MaxANPP | 1047.9 |
| Jack Pine | MaxANPP | 552.3 |
| Trembling Aspen | MaxANPP | 607.7 |
| Balsam Fir | MaxANPP | 625 |
| Yellow Birch | MaxAGB | 16720 |
| Paper Birch | MaxAGB | 11935 |
| White Spruce | MaxAGB | 14982.4 |
| Black Spruce | MaxAGB | 15830.4 |
| Tamarack Tree | MaxAGB | 15357.2 |
| Jack Pine | MaxAGB | 9095.3 |
| Trembling Aspen | MaxAGB | 10024.2 |
| Balsam Fir | MaxAGB | 10734.8 |

**Table A.3.** The life-history attributes for eight dominant tree species in the studied management units; L: longevity (years), SM: sexual maturity (years), ST: shade tolerance, SDD: seed dispersal distance (m), VRP: vegetation reproduction probability and age, PFR: post-fire regeneration strategy (Boulanger et al., 2017;Ameray et al. 2024).

| **Species** | **CODE** | **L** | **SM** | **ST** | **FT** | **SDD** | | | **VRP** | **VRP min age** | **VRP max age** | **PFR** |
| --- | --- | --- | --- | --- | --- | --- | --- | --- | --- | --- | --- | --- |
|  |  |  |  |  |  | **effective** | | **max** |  |  |  |  |
| *Abies balsamea* | BF | 150 | 30 | 5 | 1 | 25 | 160 | | 0 | 0 | 0 | none |
| *Betula alleghaniensis* | YB | 220 | 40 | 3 | 1 | 100 | 400 | | 0.1 | 10 | 180 | resprout |
| *Betula papyrifera* | WB | 140 | 20 | 2 | 1 | 100 | 1000 | | 0.5 | 10 | 70 | resprout |
| *Larix laricina* | LT | 160 | 40 | 1 | 1 | 50 | 200 | | 0 | 0 | 0 | none |
| *Picea glauca* | WS | 200 | 30 | 3 | 2 | 100 | 300 | | 0 | 0 | 0 | none |
| *Picea mariana* | BS | 220 | 30 | 4 | 2 | 80 | 200 | | 0 | 0 | 0 | serotiny |
| *Pinus banksiana* | JP | 140 | 20 | 1 | 2 | 30 | 100 | | 0 | 0 | 0 | serotiny |
| *Populus tremuloides* | TA | 130 | 20 | 1 | 2 | 500 | 5000 | | 0.9 | 10 | 130 | resprout |

**Table A.4**. Base BDA extension species mortality parameters for spruce budworm host species. SRDProb: Site Resource Dominance probability; likelihood that host species contributes to neighborhood susceptibility for outbreak initiation and spread; VulnProb: Vulnerability probability; likelihood of cohort mortality when site is attacked by BDA agent.

| **Species** | **Parameter** | **Age 1** | **Value 1** | **Age 2** | **Value 2** | **Age 3** | **Value 3** |
| --- | --- | --- | --- | --- | --- | --- | --- |
| Balsam Fir | SRDProb | 0 | 0.25 | 30 | 0.75 | 50 | 0.80 |
| Balsam Fir | VulnProb | 0 | 0.20 | 30 | 0.30 | 50 | 0.60 |
| White Spruce | SRDProb | 0 | 0.18 | 30 | 0.36 | 50 | 0.70 |
| White Spruce | VulnProb | 0 | 0.10 | 30 | 0.20 | 50 | 0.40 |
| Black Spruce | SRDProb | 0 | 0.07 | 30 | 0.15 | 50 | 0.30 |
| Black Spruce | VulnProb | 20 | 0.10 | 30 | 0.15 | 999 | 1.00 |

**Table A.5.** Annual burnt rate used for baseline and RCP climate scenarios, the historic was used as a baseline (1970-2010). Those rates were calibrated from the literature. The wildfires were interactive using 3 fire maps per period, the rates of the period 2080-2100 were used as constant after 2100.

| period | baseline | rcp26 | rcp45 | rcp85 |
| --- | --- | --- | --- | --- |
| 2010-2040 | 0.15% | 0.15% | 0.15% | 0.15% |
| 2040-2080 | 0.15% | 0.17% | 0.20% | 0.25% |
| 2080-2100 | 0.15% | 0.18% | 0.30% | 0.38% |

**Table A.6.** Base Wind extension parameters for ecoregions. Mean, Maximum and Minimum windthrow event size for each ecoregion based on historical data (1970-2010).

| **Ecoregion** | **Max size** | **Mean size** | **Min size** |
| --- | --- | --- | --- |
| eco1SALO | 111 | 35.8 | 5 |
| eco1LOSA | 1 | 0.2 | 0 |
| eco2SALO | 188 | 43.6 | 1 |
| eco3SALO | 3188 | 344.1 | 4 |
| eco3LOSA | 159 | 40.7 | 5 |
| eco4SALO | 897 | 114.5 | 1 |
| eco4LOSA | 248 | 49.6 | 1 |
| eco5SALO | 1000 | 350.6 | 2 |
| eco5LOSA | 1000 | 155.0 | 1 |
| eco6SALO | 1000 | 435.3 | 2 |
| eco6LOSA | 484 | 73.2 | 1 |
| eco7SALO | 40 | 23.0 | 1 |
| eco7LOSA | 1934 | 218.5 | 4 |
| eco8SALO | 468 | 71.6 | 5 |
| eco8LOSA | 1321 | 157.0 | 10 |
| eco9SALO | 20 | 7.4 | 1 |
| eco9LOSA | 20 | 9.9 | 1 |
| eco10SALO | 50 | 20.4 | 1 |
| eco10LOSA | 459 | 70.7 | 28 |

Table A.7 Carbon emissions (in kg CO**_2eq_** per kg of biomass processed) for five key operations involved in the forest biomass supply chain: harvesting (machinery emissions), forwarding, sawing, chipping, and pelletization. Emissions are reported for two wood density scenarios—coniferous (ρ = 384 kg/m³) and broadleaved (ρ = 500 kg/m³)—and are categorized by their applicable product pathways (sawnwood, pulp and paper, bioenergy). Fuel-based emissions apply to field operations (harvesting, forwarding), while industrial operations (sawing, chipping, pelletization) are primarily electricity-based. References indicate the source of emission factors or estimation methods.

| **Operation** | **Description** | **Applicable Products** | **kg CO_2eq_ /kg**  **(ρ = 384)** | **kg CO_2eq_/kg**  **(ρ = 500)** | **Reference** |
| --- | --- | --- | --- | --- | --- |
| Harvesting | Emissions from diesel-powered machinery (feller-bunchers, skidders, delimbers) used to cut and collect trees in the forest. | All | 0.00597 | 0.00458 | Kärhä et al. (2024) |
| Forwarding | Emissions from diesel-powered forwarders used to transport felled logs from the stump to the roadside landing for final transporting to the transformation facility. | All | 0.00480 | 0.00370 | Kärhä et al. (2024) |
| sawnwood production | Emission factor estimated based on total energy used to process logs into planed, kiln-dried softwood lumber. The operation includes the Sawing (including handling, debarking, sorting), Kiln Drying, Surface Planing and Packaging. | Sawnwood | 0.0972 | 0.0744 | NRcan, 2010 |
| Chipping | Emissions from converting biomass into wood chips using electricity-powered chippers. This material is used for pulp&paper or bioenergy. | Pulp & Paper, Bioenergy | 0.0174 | 0.0134 | Laganière et al. (2017) |
| Pelletization (Drying, Milling, Compacting) | Emissions from preparing wood for bioenergy and pulp and paper pellets. Includes drying biomass to ~8–12% moisture, grinding it into powder, and compressing it through a pellet press. Most energy-intensive step. We used the highest value found in the literature 10.45 kg CO₂/GJ. | Pulp & Paper, Bioenergy | 0.2393 | 0.1839 | Laganière et al. (2017) |

Figure A.1 Projections of temperature, precipitation, and atmospheric CO₂ concentration under different climate scenarios (RCP26, RCP45, and RCP85) based on the CanESM2 model, and the IPCC database for CO₂. The gray bands represent the historical period from 1981 to 2010, and used a baseline.


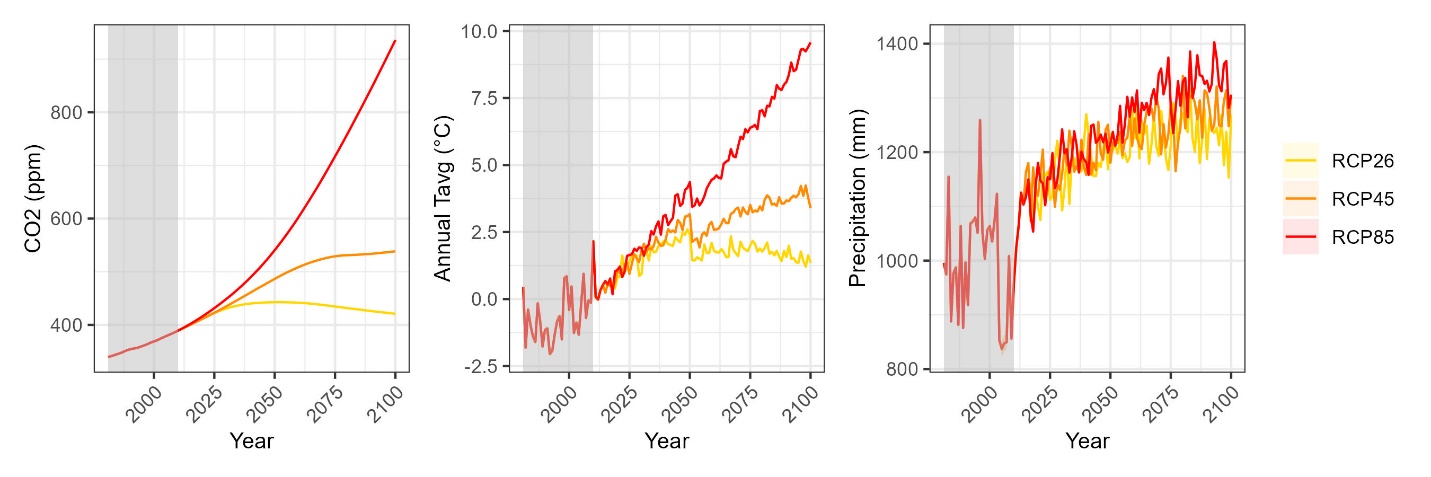


Figure A.2 The empirical proportion of the area affected (mortality and defoliation) relative to the total forest area based on aerial photographs in the Côte-Nord regions during the 1967-1988 outbreak compared to the average in Quebec boreal forest. The obtained simulated curves represent the first outbreak under current climate conditions. We maintain a consistent duration of mortality across all our simulations.


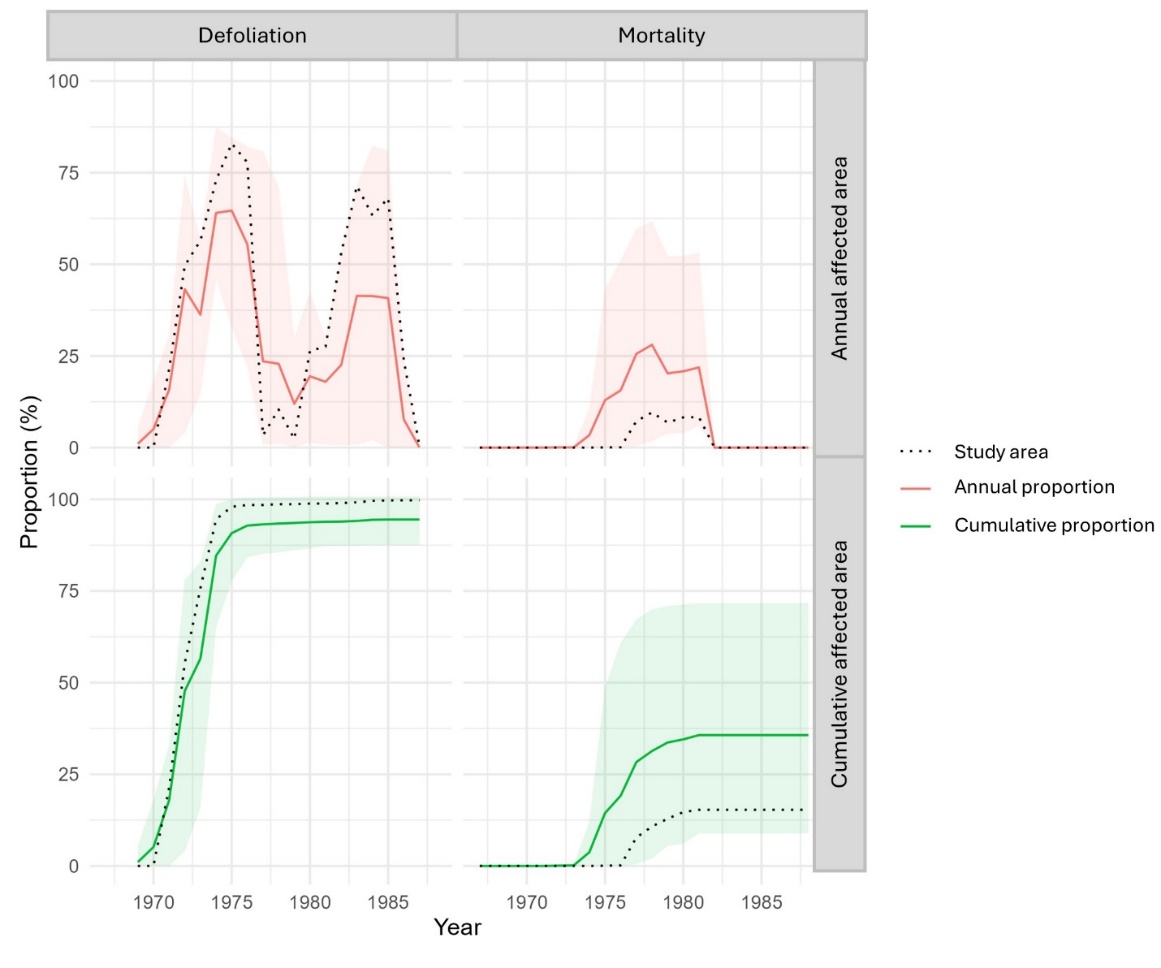


**Figure A.3** Example of the spatial spread of the first spruce budworm outbreak (2010-2017) under current conditions. An ecoregion modifier was applied exclusively to the first outbreak event to boost spread toward the northern region. However, because the initial outbreak epicenter is stochastically determined, the outbreak may still occur in southern areas, though with a lower probability compared to northern regions. The accumulative raster presents the total affected area by mortality.


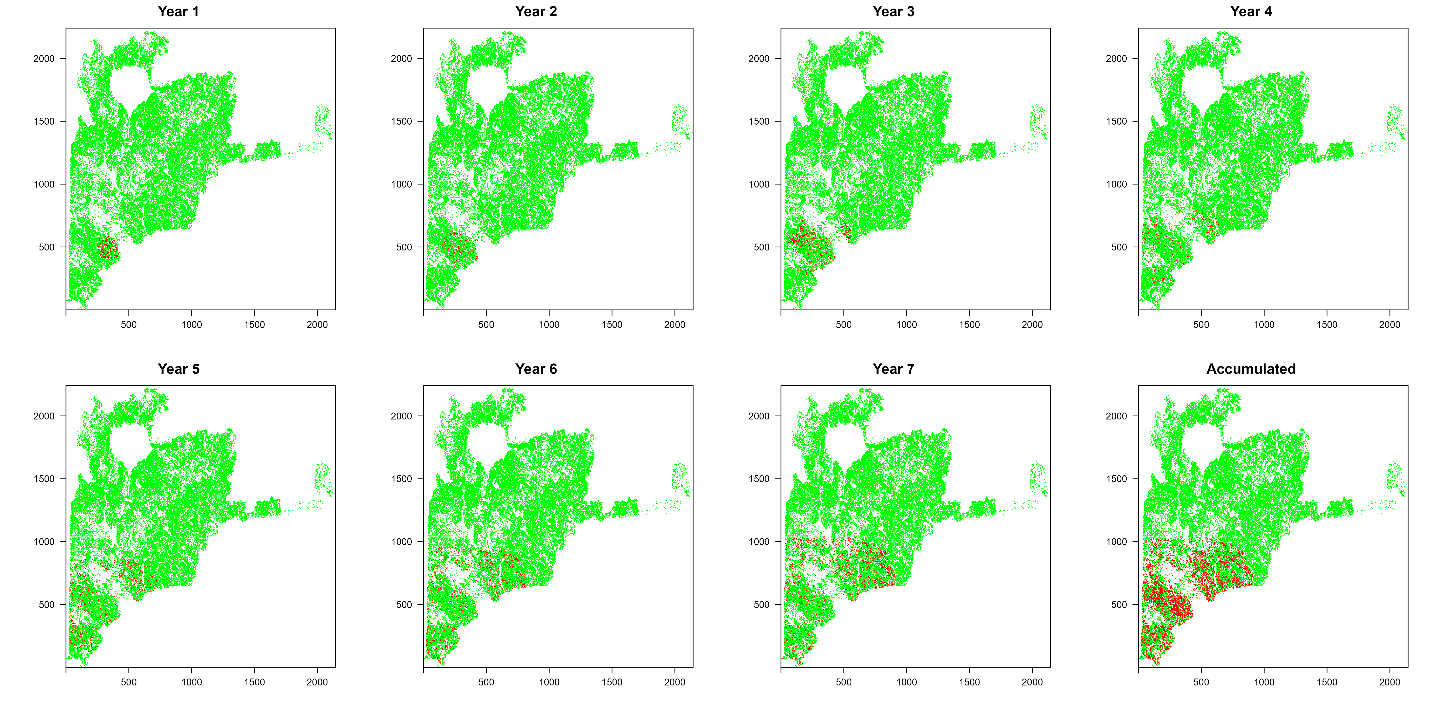


Figure A.4 Landscape-scale model validation of aboveground biomass (AGB). (A) Comparison of mean AGB (t ha⁻¹) between empirical observations and model simulations (error bars show 95% confidence intervals); RMSE is reported for the overall fit, and the mean difference (Δ) is indicated. (B) Spatial distribution of land types across the study landscape in eastern boreal Québec, Canada. (C) Quantile-based comparison (Q10–Q90) highlighting agreement and departures across the biomass distribution. (D) Mean biomass by land type for empirical and simulated data (error bars show within–land type variability), illustrating spatial heterogeneity in model performance across ecological units.


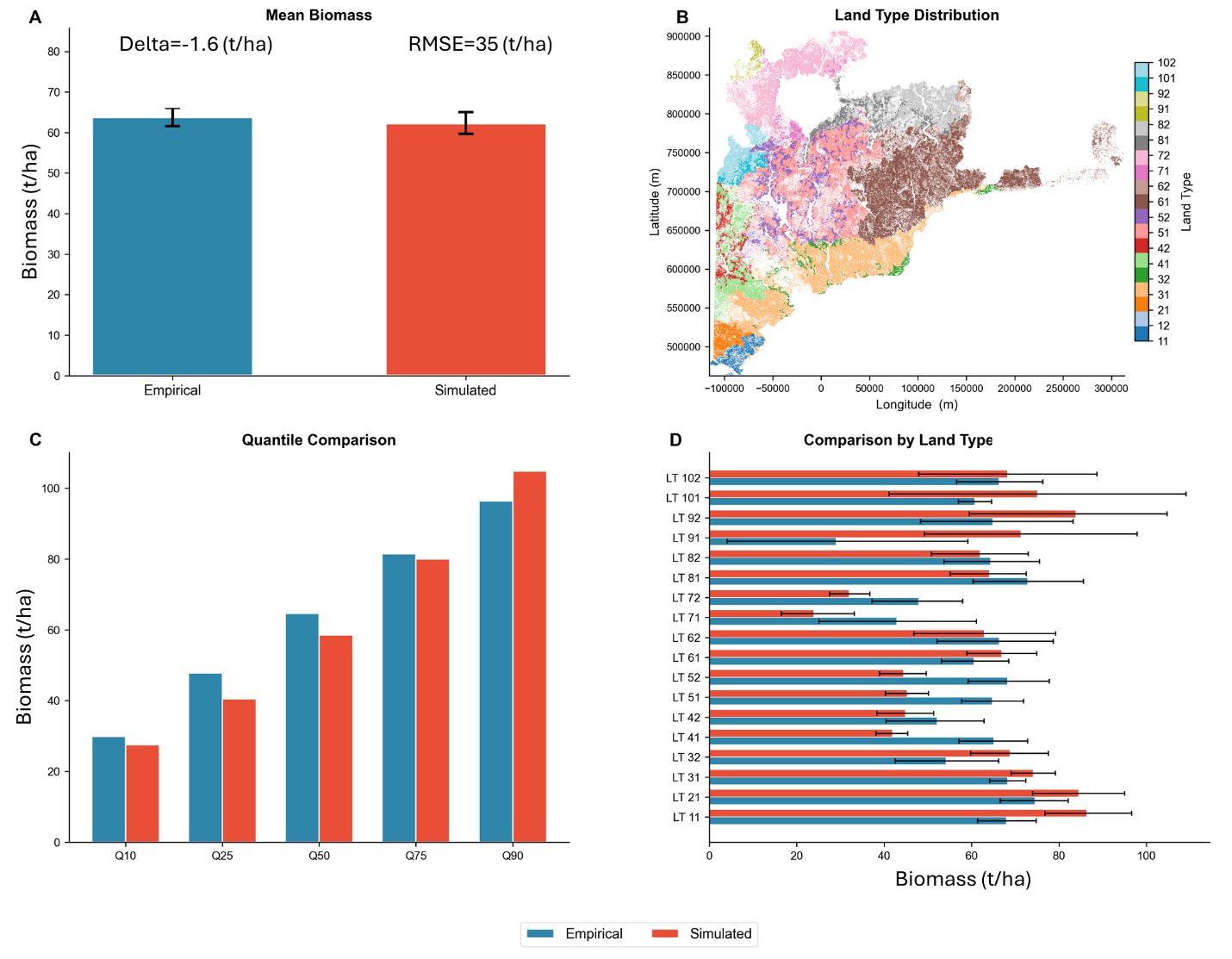


Figure A.5 Forest age distribution under different climate change pathways. Extreme climate change scenarios may lead to an increase in young forest cover and a decline in old-growth forest occupancy.


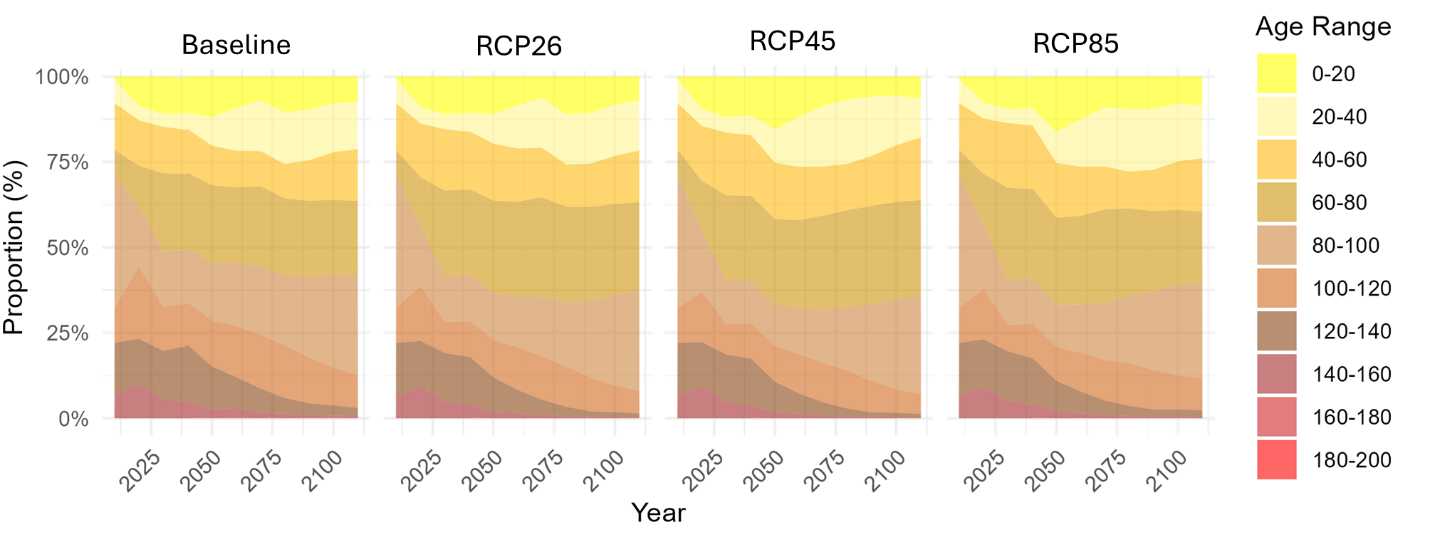


Figure A.6 Species abundances (tC ha^-1^ yr^-1^) under different climate change pathways. No differences in species abundance were observed among management scenarios, as salvage biomass operations target only dead biomass resulting from the outbreak. The first outbreak has considerably reduced the abundance of balsam fir.


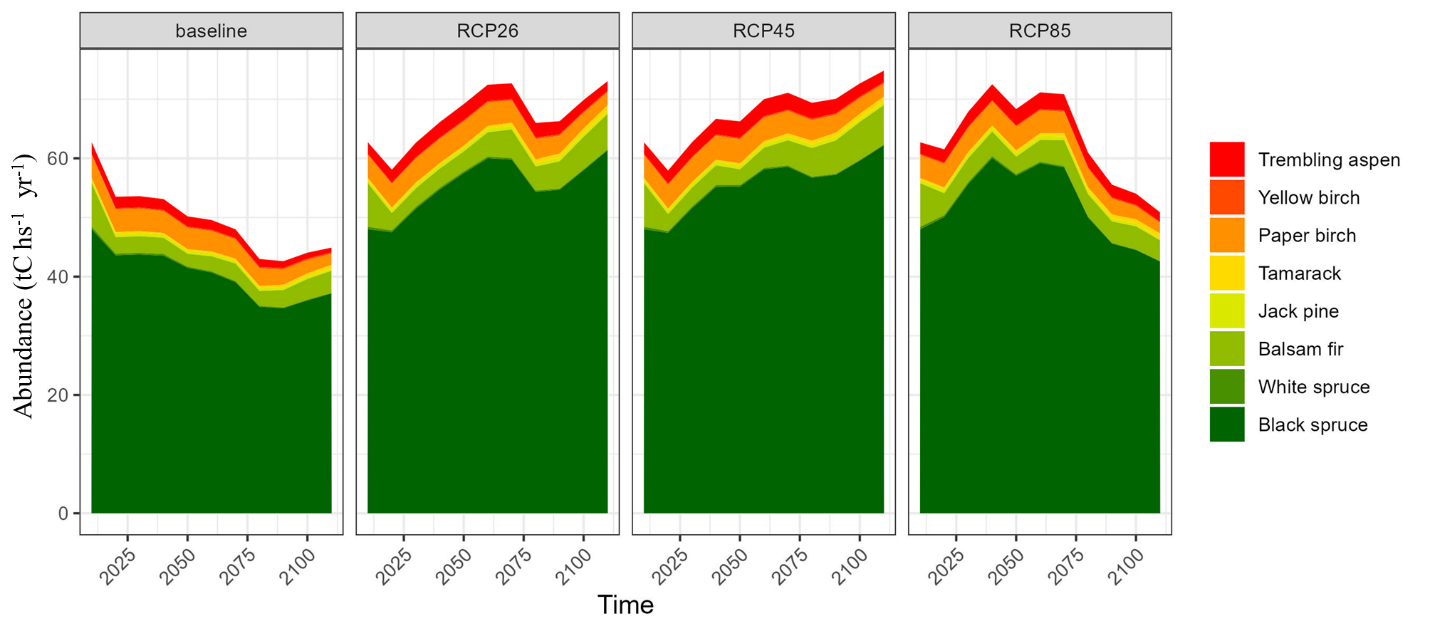


Figure A.7 Temporal dynamics of NEP and NBP differences (delta) between management scenarios S1 to S3 and the reference (S0), under various climate change pathways (baseline, RCP2.6, RCP4.5, RCP8.5). Shaded gray bands indicate periods of spruce budworm (SBW) outbreak-induced tree mortality. There is no effect of salvage biomass on NPP.


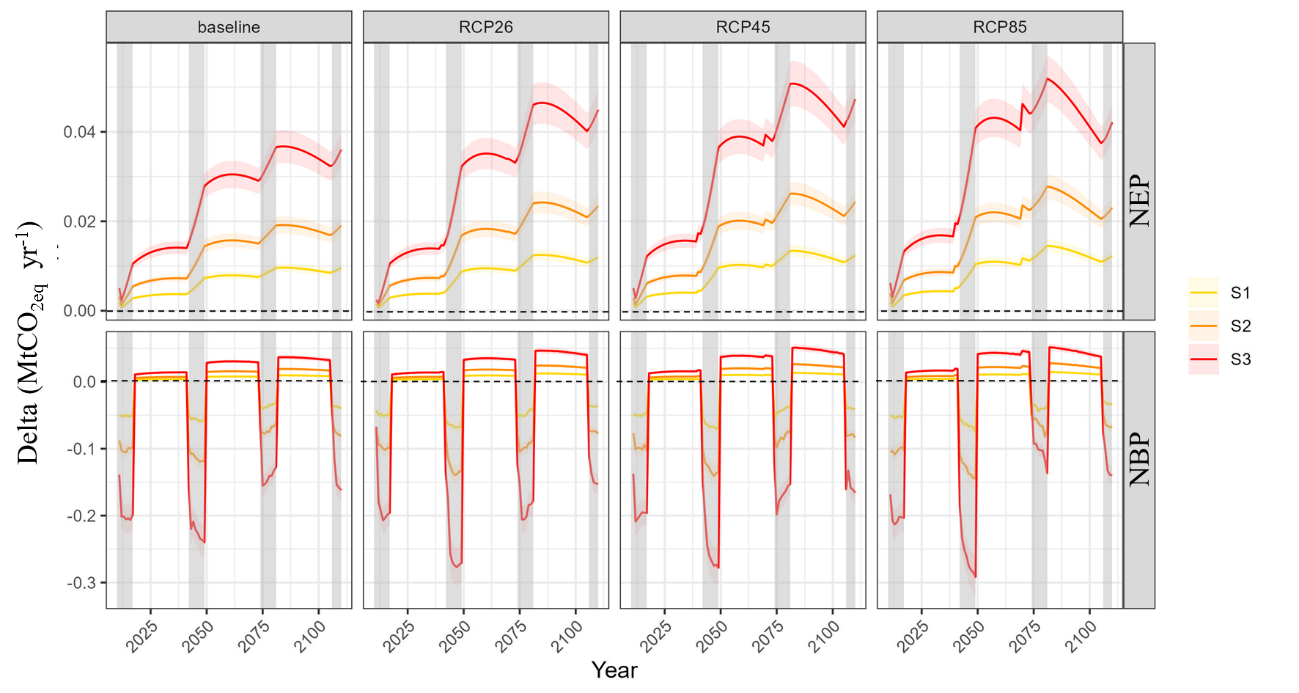


Figure A.8 Avoided carbon emissions from decomposition (top row) and fire (bottom row) at the ecosystem scale under four climate change pathways (baseline, RCP2.6, RCP4.5, RCP8.5) and four management scenarios: reference (S0) and three salvage biomass harvesting intensities (S1–S3). Values represent differences in annual emissions (MtCO₂ eq yr⁻¹) relative to the reference scenario (S0). Negative values indicate a reduction in emissions due to salvage harvesting. Grey vertical bands denote periods of simulated spruce budworm outbreaks.


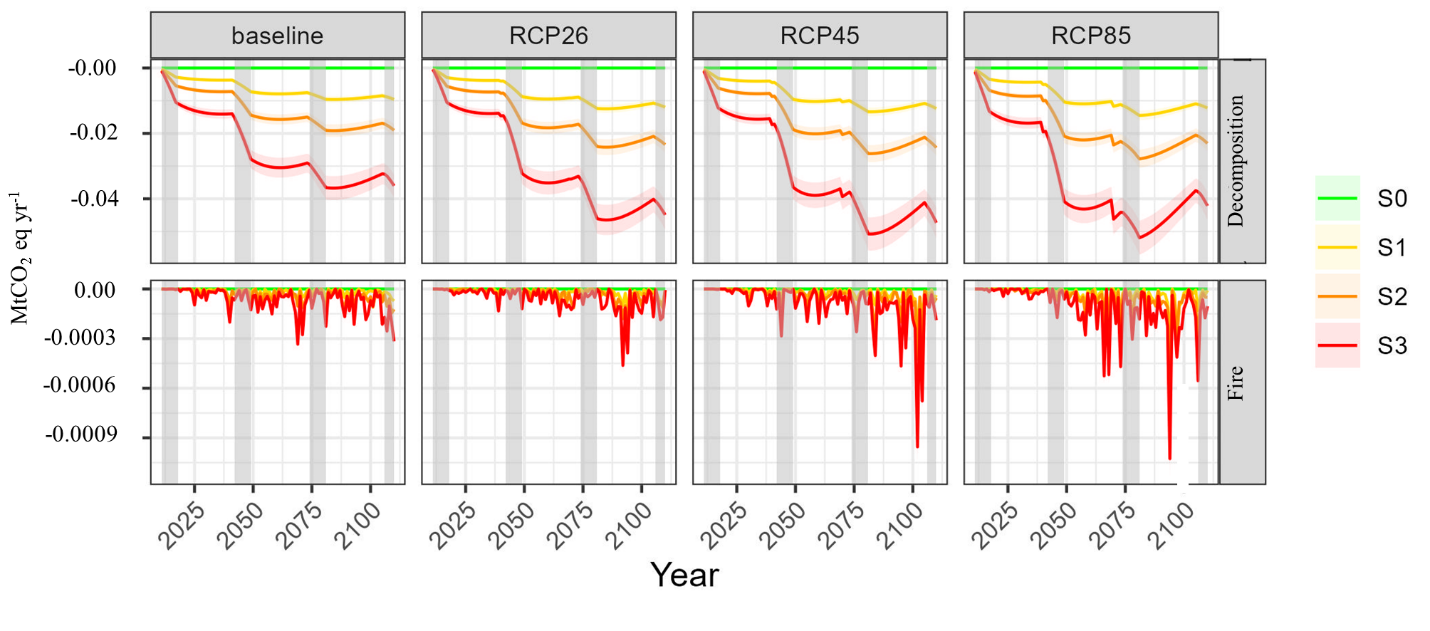


Figure A.9 Temporal dynamics of carbon remaining and emissions from harvested wood products (HWP) by product category (Sawnwood, Pulp & Paper, and Bioenergy) over 100 years under different climate change pathways (Baseline, RCP2.6, RCP4.5, RCP8.5) and current reference scenario (S0). The half-life used for sawnwood under S0 is 35 years.


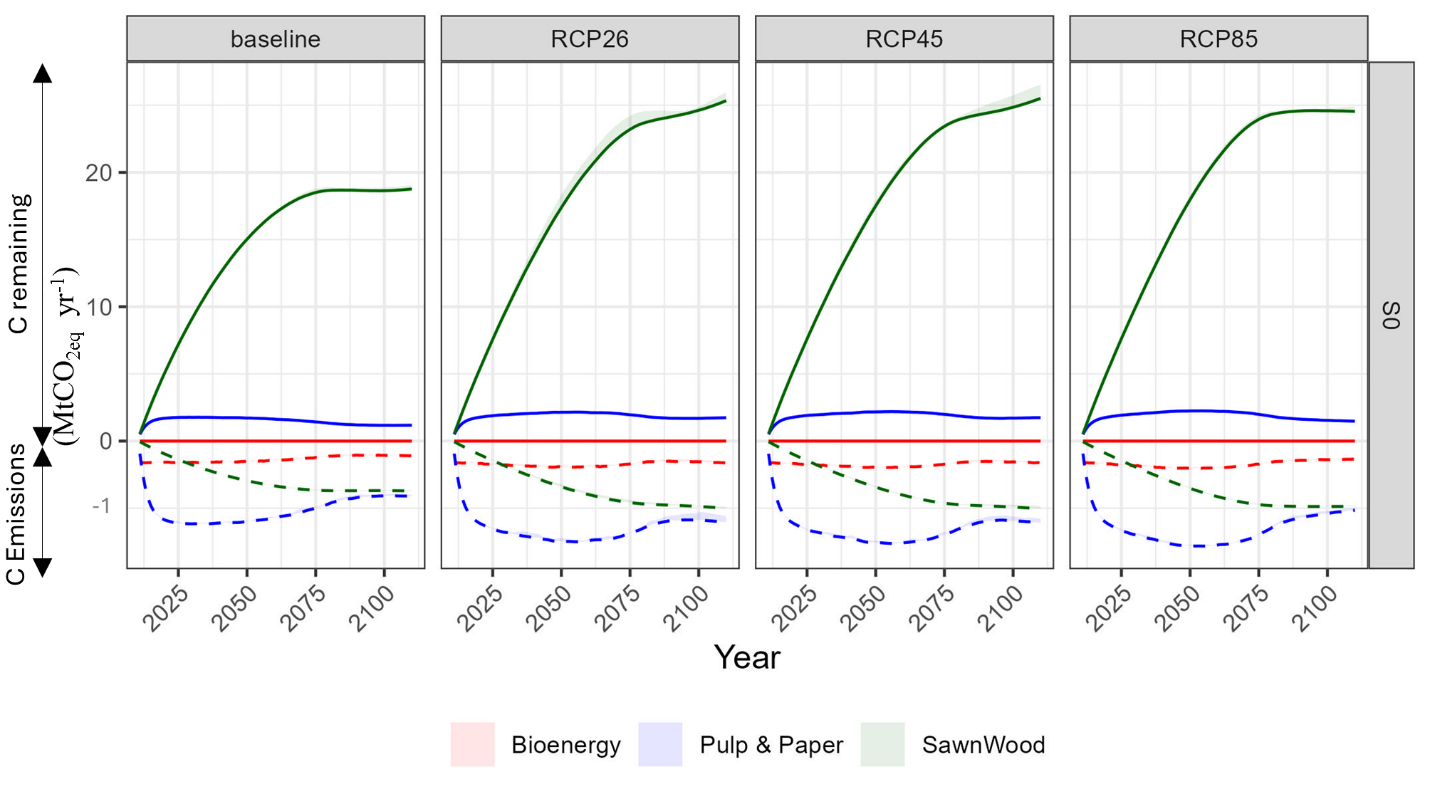


Figure A.10 the magnitude of change (Δ) in carbon remaining in harvested wood products and their associated emissions expressed on MtCO2eq yr^-1^ (combustion for bioenergy and decomposition of swanwood and pulp&paper) between salvaged biomass scenarios (S1, S2, S3) and reference scenario (S0).


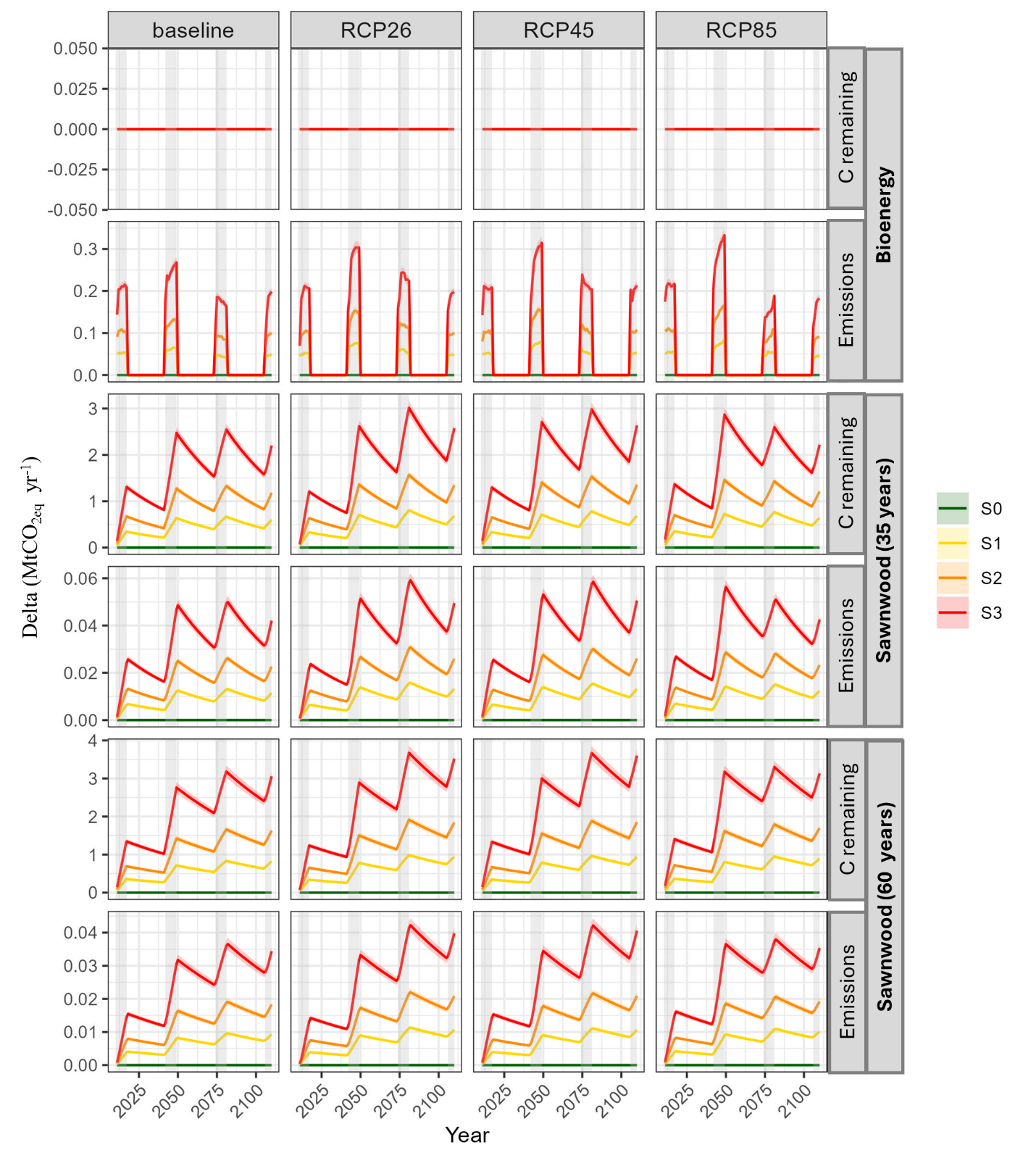


Figure A.11 Delta in total carbon emissions (combustion and decomposition) from harvested wood products (HWP) and forest ecosystems under salvage biomass scenarios (S1–S3) compared to the no-salvage scenario (S0), across four climate pathways (baseline, RCP2.6, RCP4.5, RCP8.5) and three different additional biomass uses (bioenergy, sawnwood with half-life 35 years and sawnwood with half-life 60 years). Gray bands indicate periods of spruce budworm (SBW) outbreak-induced tree mortality.


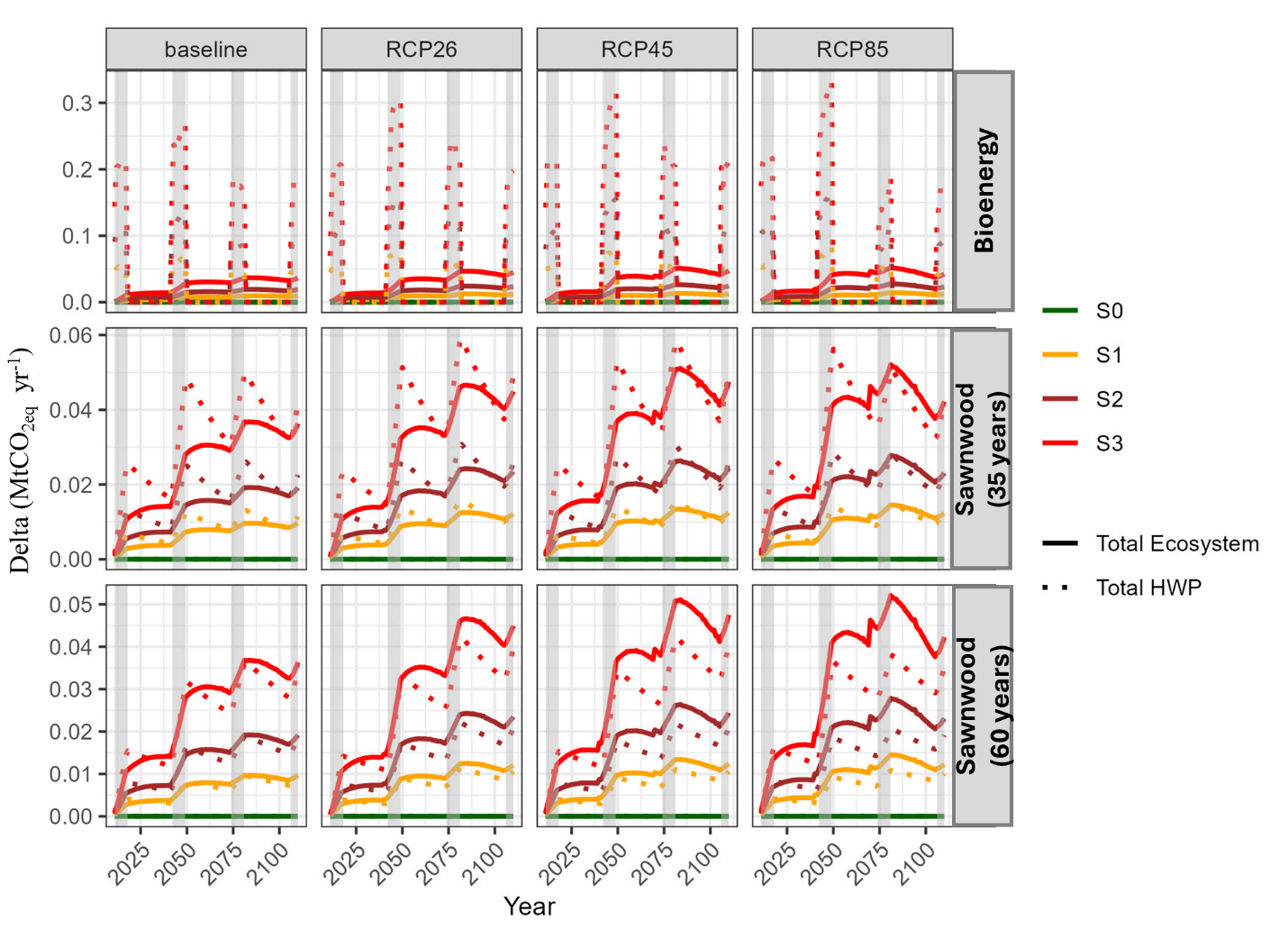


Figure A.12 The differences (Δ) between salvage biomass scenarios and the reference (S0) for : Annual carbon remaining (Cr) flux after accounting for decomposition and combustion emissions from bioenergy use; Carbon emissions associated operation of wood processing (Harvest, forwarding, transport, sawing, chipping, palletization); and Net Sector Production (NSP), integrating all gains and losses in the forest sector (all variables expressed in MtCO2eq yr⁻¹). Shaded gray bands represent periods of spruce budworm (SBW) outbreak-induced tree mortality (2010–2017, 2042–2049, 2074–2081, and 2106–2110).


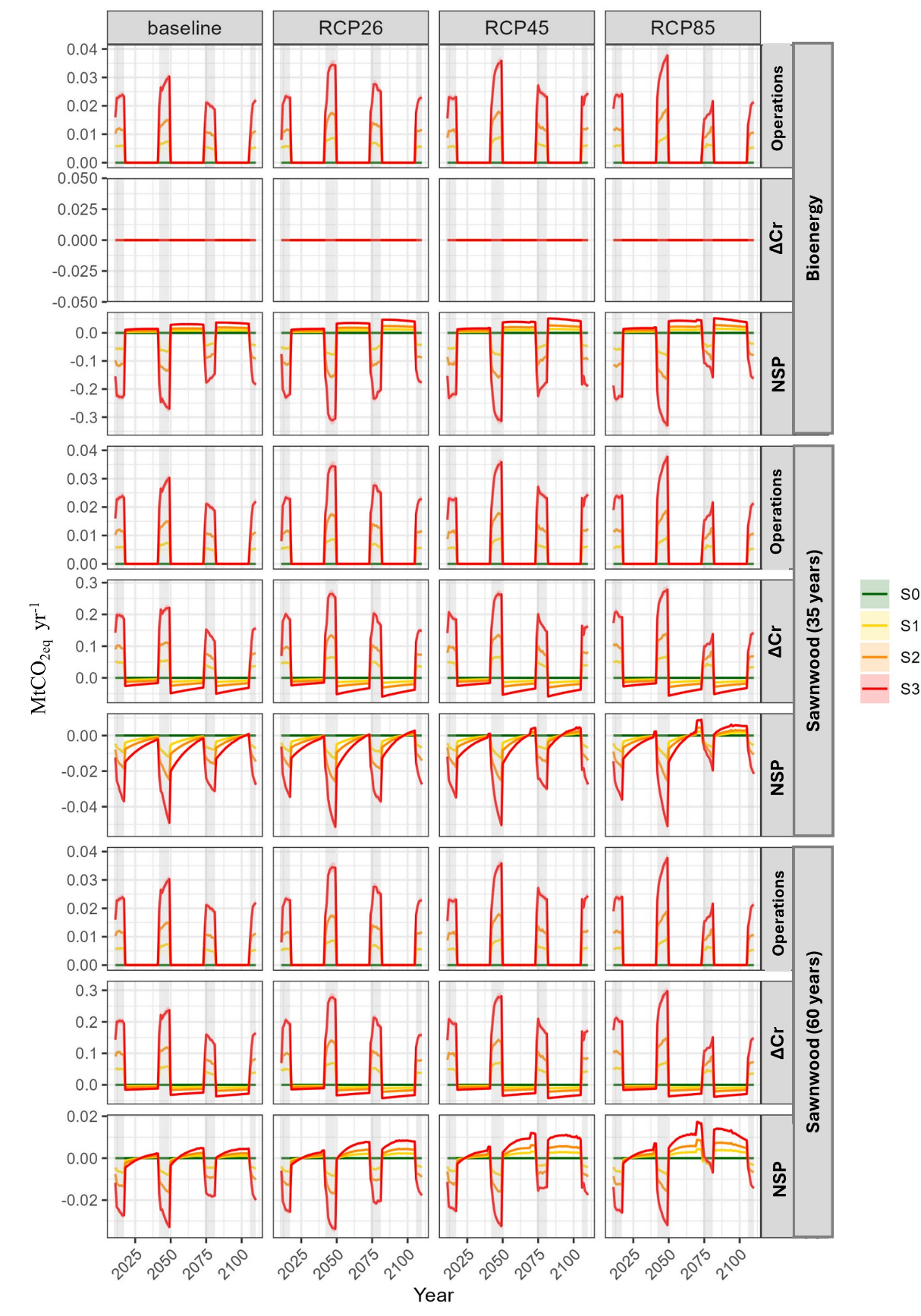

Supplement: Supplementary file 1 — Supplementary file1 (DOCX 7758 KB) [file 10980_2026_2376_MOESM1_ESM.docx]
